# Effects of RNA methylation N6-methyladenosine regulators on malignant progression and prognosis of melanoma

**DOI:** 10.1186/s12935-021-02163-9

**Published:** 2021-08-26

**Authors:** Jinfang Liu, Zijian Zhou, Ling Ma, Chujun Li, Yu Lin, Ting Yu, Ji-Fu Wei, Lingjun Zhu, Gang Yao

**Affiliations:** 1 Department of Plastic and Burns Surgery, The First Affiliated Hospital of Nanjing Medical University, 300 Guangzhou Road, Nanjing, 210029 China; 2 Department of Urology, The First Affiliated Hospital of Nanjing Medical University, 300 Guangzhou Road, Nanjing, 210029 China; 3 Department of Oncology, The First Affiliated Hospital of Nanjing Medical University, 300 Guangzhou Road, Nanjing, 210029 China; 4 Research Division of Clinical Pharmacology, The First Affiliated Hospital of Nanjing Medical University, 300 Guangzhou Road, Nanjing, 210029 China

**Keywords:** Melanoma_1_, m6A RNA methylation_2_, Prognostic signature_3_, TCGA_4_, IGF2BP3_5_

## Abstract

**Background:**

Melanoma is an extremely aggressive type of skin cancer and experiencing a expeditiously rising mortality in a current year. Exploring new potential prognostic biomarkers and therapeutic targets of melanoma are urgently needed. The ambition of this research was to identify genetic markers and assess prognostic performance of N6-methyladenosine (m6A) regulators in melanoma.

**Methods:**

Gene expression data and corresponding clinical informations of melanoma patients as well as sequence data of normal controls are collected from The Cancer Genome Atlas (TCGA) and the Genotype-Tissue Expression (GTEx) databases. Quantitative real-time PCR (qRT-PCR) analysis was carried out to detect the RNA expression of IGF2BP3 in A375 cell line, melanoma tissues, and normal tissues. Western blot, cell proliferation, and migration assays were performed to assess the ability of IGF2BP3 in A375 cell line.

**Results:**

Differently expressed m6A regulators between tumor samples and normal samples were analyzed. A three-gene prognostic signature including IGF2BP3, RBM15B, and METTL16 was constructed, and the risk score of this signature was identified to be an independent prognostic indicator for melanoma. In addition, IGF2BP3 was verified to promote melanoma cell proliferation and migration in vitro and associate with lymph node metastasis in clinical samples. Moreover, risk score and the expression of IGF2BP3 were positively associated with the infiltrating immune cells and these hub genes made excellent potential drug targets in melanoma.

**Conclusion:**

We identified the genetic changes in m6A regulatory genes and constructed a three-gene risk signature with distinct prognostic value in melanoma. This research provided new insights into the epigenetic understanding of m6A regulators and novel therapeutic strategies in melanoma.

**Supplementary Information:**

The online version contains supplementary material available at 10.1186/s12935-021-02163-9.

## Introduction

Melanoma is a highly aggressive form of skin cancer, forming by malignant transformation of melanocytes [1], frequently leading to metastasis with a mortality rate exceeds 80% [2–5]. The absence of accurate metastatic diagnostics forces it unfeasible to be treated by surgery operations briefly [5, 6]. Many risk factors for melanoma have been found out, including environmental and genetic factors [7, 8]. In the last decade, increasing researches have concentrated on the multiple molecular pathways that refer to melanoma pathogenesis, genetic, and especially epigenetic events [9]. The accumulation of genetic and epigenetic alterations results in a multistep process which includes the activation of oncogenes and the inactivation of tumor suppressor genes, eventually leading to the development of melanoma.

M6A RNA modification is a considerably dynamic and reversible process modulated by methyltransferases and demethylases [10]. Notably, m6A regulating proteins which include "writers", "erasers" and "readers" (WERs) were reported with important effects in tumor initiation and progression [11]. The abnormal methylation of m6A mRNA has been reported to be associated with poor prognosis in breast cancer, bladder cancer, head and neck squamous cell carcinoma, glioblastoma, and colorectal cancer patients [12–16]. However, some studies demonstrated the antitumor effect of m6A. For instance, HNRNPC was an essential participant in the malignant progression of glioblastoma and patients with high gene expression of HNRNPC were reported to have a good prognosis [17]. VIRMA was associated with better OS in papillary thyroid carcinoma [18, 19]. Moreover, for the prognostic value in melanoma, some m6A WERs play significant roles in the malignant progression and prognostic parts of uveal melanoma [20]. Also, m6A genetic alterations have been found to be closely related to cutaneous melanoma patients’ survival outcomes [21]. However, systematic research on the prognostic value of m6A in melanoma remains scarce.

In this study, we systematically evaluated the association between the gene expressions of the m6A WERs and melanoma, analyzed the association of the m6A WERs and overall survival (OS) of melanoma patients, and constructed a melanoma prognosis signature based on the selected m6A regulators for melanoma patients.

## Materials and methods

### Dataset acquisition

The data of clinical information and gene expression profiles of melanoma patients and normal controls in this paper are mainly downloaded from TCGA (https://portal.gdc.cancer.gov/) and GTEx (https://toil.xenahubs.net/download/GTEX_phenotype.gz) datasets. TCGA-melanoma and GTEx gene expression data were analyzed by the same library preparation and sequencing platform for the minimized potential batch effects [22]. A total of 471 tumor samples and 1 normal sample (from the TCGA) and 812 normal skin samples (from the GTEx) were statistically analyzed for follow-up.

### Selection and differential expression analysis of m6A WERs regulators

In a summing up of the latest published review considering m6A regulators in human cancers [11], we collected twenty m6A WERs regulators (WTAP, RBM15B, RBM15, KIAA1429, METTL3, METTL14, METTL16, IGF2BP1, IGF2BP2, IGF2BP3, ZC3H13, CBLL1, HNRNPC, YTHDC1, YTHDC2, YTHDF1, YTHDF2, YTHDF3, FTO, and ALKBH5) with available expression and clinical data in TCGA and GTEx datasets. Subsequently, utilizing the “Limma” R package for identifying differentially expressed genes (DEGs), these m6A WERs regulators expression levels between tumor and normal sample groups were compared respectively. Next, the expression level and different clinical characteristics (AJCC stage) of these m6A genes in melanoma were also compared. Ultimately, for visualizing the differential expression patterns of m6A WERs regulators, the “pheatmap” and “violinplot” R package were exerted.

### Construction of PPI network and correlation analysis

Using the STRING online database (http://string-db.org/) and Cytoscape software (version 3.8.2), the protein–protein interaction (PPI) network was constructed and reprocessing. Meanwhile, the association among those m6A regulators was further investigated by the co-expression correlation analysis using the “corrplot” R package.

### Construction and validation of prognostic signatures

The samples with entire survival and clinical information were randomly divided by the “caret” package into two groups (the training and testing cohort). Then univariate Cox regression analysis was applied to denote the prognostic value of the m6A regulators’ expression in the training cohort. Next, m6A genes significantly associated with OS in univariate analysis were followingly chosen to establish an m6A-related risk signature by LASSO Cox regression algorithm [23]. As the result above, three m6A regulators (IGF2BP3, METTL16, and RBM15B) were figured out by the minimum mean cross-validated error with their corresponding coefficients. And the optimal penalty parameter related to the minimum tenfold cross validation was selected within the training cohort. Risk Score is equal to the result of (λ1 * expression of A) + (λ2 * expression of B) + (λ3 * expression of C) + … + (λn * expression of N), in which “λ” represents the regression coefficient of each gene. Based on the median risk score values, the melanoma patients in the training and testing cohorts were divided into low-risk and high-risk subgroups. For evaluating the differential OS between the high-risk and low-risk subgroups, the Kaplan–Meier method was utilized. The prediction efficiency of the three-gene risk signature was analyzed by the ROC analysis. The three-gene risk signature was validated by the Kaplan–Meier curve and ROC curve in the validation group.

### Independent prognostic ability of the three hub genes signature

It is significant to identify whether clinicopathologic features (age, gender, and AJCC TNM stage) and risk score were independent prognostic factors for melanoma patients, univariate and multivariate Cox regression analyses were exerted both in the training and testing cohort. The multivariate Cox regression analysis included all knew prognostic factors (age, gender, AJCC TNM stage, and risk score) for the outcome of interest to adjust confounding and helped achieve an adjusted analysis. The OS difference stratified by age, gender, and AJCC stage between the high-risk and low-risk subgroups were explored by the Kaplan–Meier method.

### Validation of the three hub gene signatures using qRT-PCR

The tumor and paired normal samples were acquired from melanoma patients at the First Affiliated Hospital of Nanjing Medical University and stored in liquid nitrogen. This study was approved by the Institute Ethics Committee of the hospital, and the written informed consent was obtained from all melanoma patients. The total RNA of samples was extracted utilizing Trizol reagent (Invitrogen, USA). The cDNA for clinical samples and A375 cell line were reverse transcribed by HiScript II (Vazyme, China) and conducted with the SYBR-Green method. The sequences of the primers performed in this research were as follows:

- β-actin-F: 5′-TCACCCACACTGTGCCCATCTACGA-3′
- β-actin-R: 5′-CAGCGGAACCGCTCATTGCCAATGG-3′
- RBM15B-F: 5′-TTGTCTCCAACCTTCCGTAGT-3′
- RBM15B-R: 5′-CCAGATCAGAGAGGTGGTGTAG-3′
- IGF2BP3-F: 5′-AGTGCCGACAGCATTGGTG -3′
- IGF2BP3-R: 5′-GGAGCAGAGGTATCATAGGAAGC-3′
- METTL16-F: 5′-TTGTCTCCAACCTTCCGTAGT-3′
- METTL16-R: 5′-CCAGATCAGAGAGGTGGTGTAG-3′.

### Cell culture

The Cell Bank of the Chinese Academy of Sciences provided the human melanoma A375 cell line and confirmed the cells using short tandem repeat profiling. The A375 cell line was cultured in RPMI-1640 (Gibco, USA) with 10% foetal bovine serum (Gibco, USA) and 1% penicillin/streptomycin (Invitrogen, USA). Cells were cultured in a humidified incubator at 37 ℃ in 5% CO2.

### Lentivirus transduction

The lentivirus construction of IGF2BP3 knockdown and overexpression was provided by OBIO (Obio Technology Corp, China). Briefly, the A375 cells were plated at 50% confluence in 6 wells dishes. Then the A375 cells were infected with IGF2BP3 overexpression, scramble control, knockdown lentivirus, and negative control lentivirus (termed as IGF2BP3, NC, shIGF2BP3-1, shIGF2BP3-2, and shNC). The pools of stable transductions were accomplished by puromycin (4 μg/ml).

### Western blot

Cells treated with lentivirus were lysed with RIPA (Beyotime, China) buffer containing protease inhibitors (Sigma-Aldrich). The 10% SDS-PAGE gels separate total protein lysates and polyvinylidene fluoride (PVDF) membranes (Millipore, USA) transferred the protein lysates. The anti-IGF2BP3 antibody (1:1000, Proteintech, China), anti-β-Actin antibody (1:1000, Proteintech, China), and peroxidase (HRP)-conjugated secondary antibody (1:5000, Proteintech, China) incubated the membranes. After washing, signals were developed utilizing the chemiluminescence system (Bio-Rad, USA) and processed by Image Lab Software (NIH).

### Cell proliferation assay

For exploring the proliferation of A375 cells, cell Counting Kit‐8 (CCK‐8; Beyotime, China) was performed. Briefly, the cells were taken in the logarithmic growth phase and reseeded into 96‐well plates (1 × 10^3^ per well) after digestion with 0.25% trypsin (Gibco, USA). Followingly, the cells were incubated for 48 h at 37 °C with 5% CO2. The cells were maintained without light in the incubator after addition of CCK‐8 solution. The microplate reader (Varioskan Flash, CA) was exerted to test the optical density of every well at 450 nm.

### Colony formation assay

The cell suspension of a single-layer culture cell in the logarithmic growth phase was diluted by multiple gradients and inoculated the culture dish with a suitable cell density (based on the proliferation rate). Next, the supernatant was discarded and cells were washed twice with PBS, added 5 mL of pure methanol or 1:3 acetic acid/methanol and fixed for 15 min at room temperature. Then, the fixative solution was removed, an appropriate amount of Giemsa stain was added and the staining solution was applied for 10–30 min. At last, we inverted the plate, overlaid a grid of transparencies, and counted the clones directly by the naked eye.

### EdU assay

EdU staining was established by the BeyoClick™ EdU Cell Proliferation Kit with Alexa Fluor 594 (Beyotime, China). A375 cells were washed with PBS. Fresh RPMI-1640 (Gibco, USA) was added, and then, 10 µM EdU was added to the plate. The cells were incubated for 2 h at 37 °C/5% CO2. After the incubation, the cells were washed with PBS to remove the RPMI-1640 and the free EdU probe. The cells were then fixed in 4% paraformaldehyde for 30 min at room temperature, then stained with DAPI for 3 min. After another wash in PBS, the cells were observed under an inverted microscope.

### Wound healing assay

A375 cells were inoculated into 6-well plates and cultured until > 95% confluence. Then the cell layer was lightly scraped with a sterile plastic tip through the central axis and nonadherent cells and debris were washed away and the media replaced with serum free media for overnight incubation. Quantification of cell motility by calculating the distance between the invading fronts of cells in four randomly chosen microscopic fields (× 100) for each condition and time point (0, 24 h).

### Statistical analysis

Except for the aforementioned analysis by R software (version 4.0.1; R: https://www.r-project.org), all other statistical data and figures were analyzed by SPSS 26.0 (IBM, USA) and GraphPad Prism 8.0 (GraphPad Software, USA). The associations between m6A expressions and different clinicopathological features were analyzed with the chi-square test or Fisher exact test. For statistical comparison between two independent experimental groups (Student’s t-test) and among more than two experimental groups (ANOVA test), appropriated statistical tests were assayed. *P* < 0.05 was considered to be statistically significant.

## Results

### The landscape of m6A RNA methylation regulators in melanoma

**Fig. 1:**
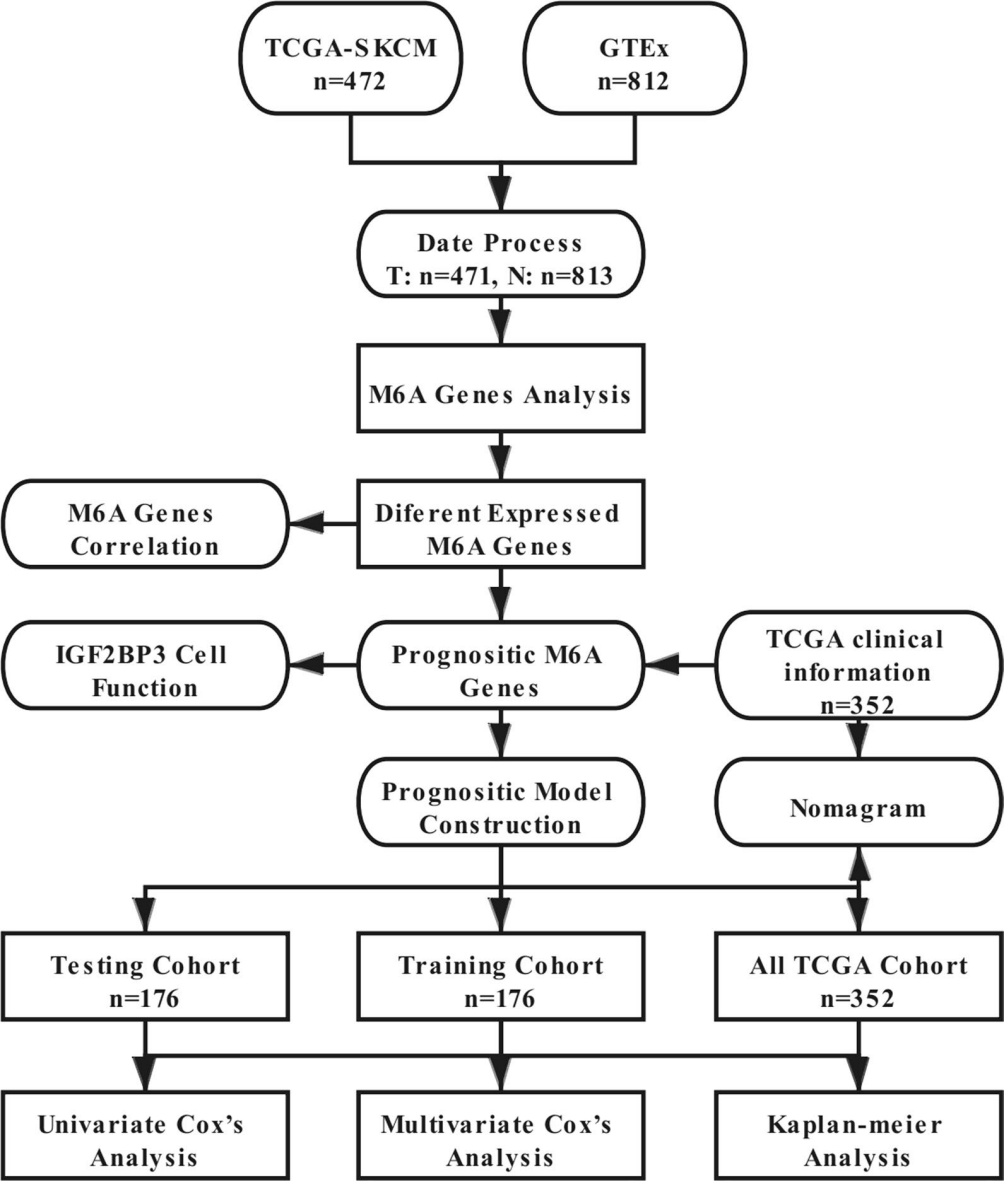
The flow chart of the analysis procedure in identifying an m6A-related prognostic signature (T: Tumor, N: normal)

**Fig. 2:**
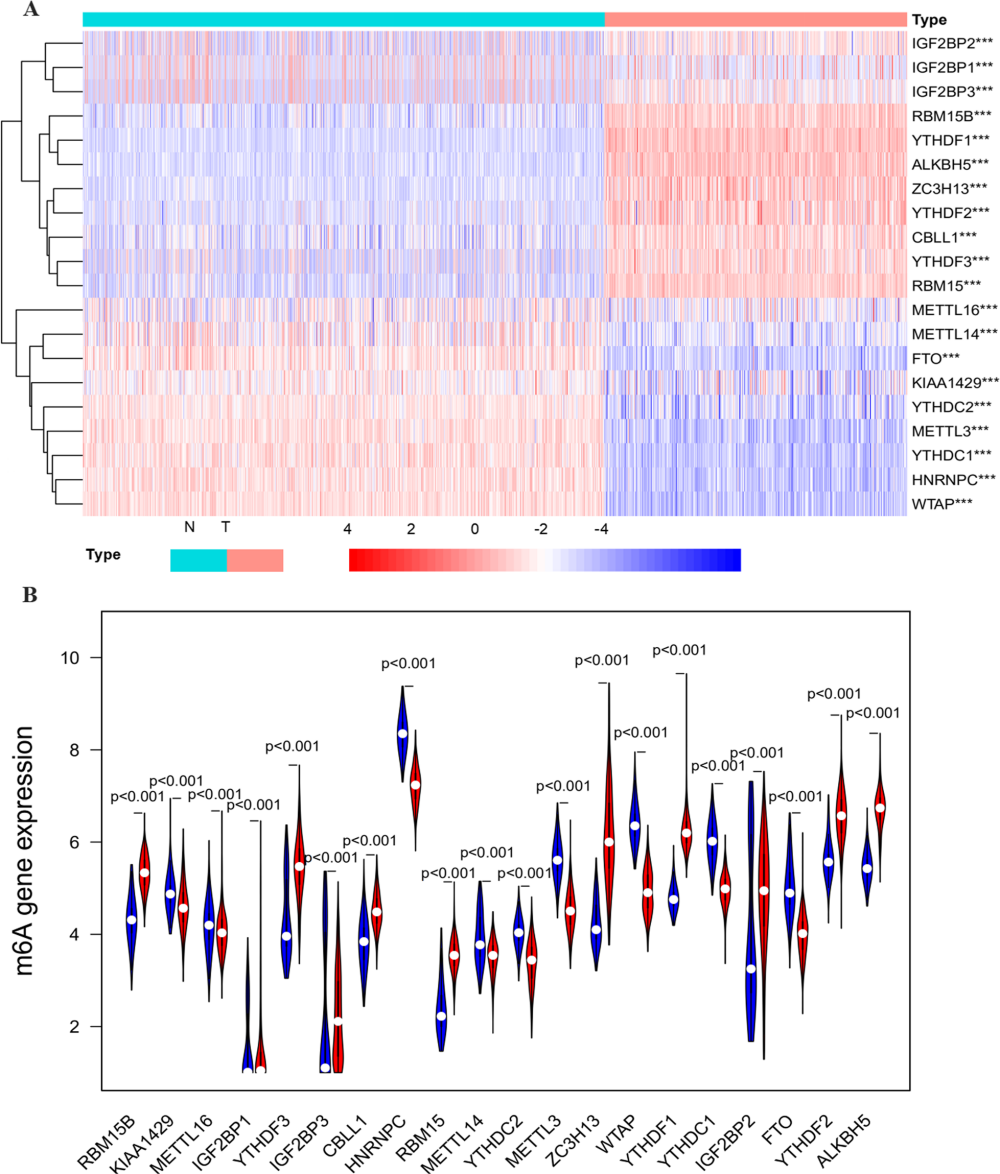

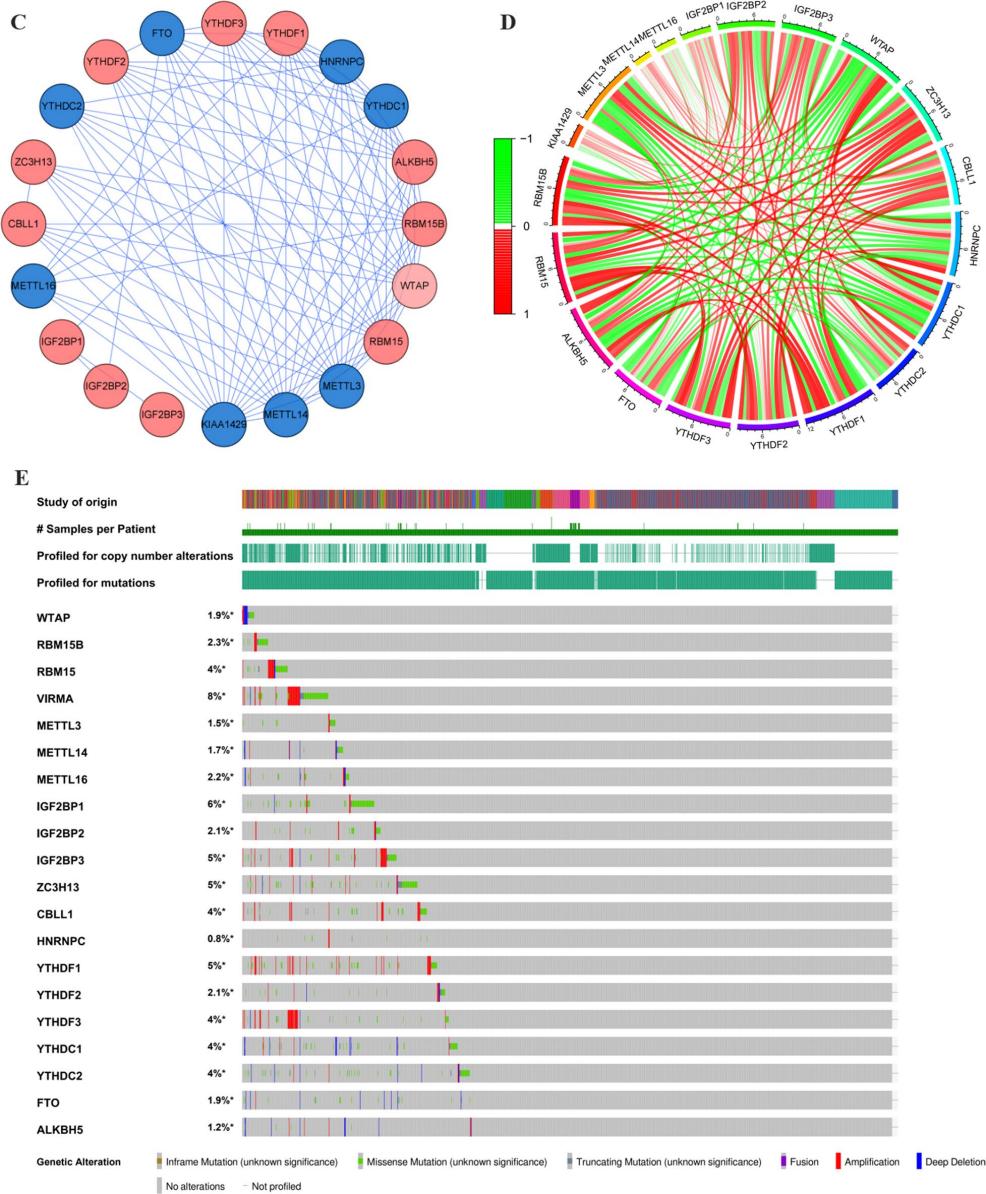
The expression pattern of m6A RNA methylation regulators in the TCGA melanoma cohort. **A** Heatmap visualizing the expression levels of m6A RNA methylation regulators in tumor samples and normal samples. **B** Vioplot visualizing the differentially expressed m6A RNA methylation regulators in TCGA. (*P < 0.05, **P < 0.01, ***P < 0.001). **C** The PPI network of the twenty selected m6A RNA methylation regulators. **D** The Pearson correlation analysis of the twenty selected m6A RNA methylation regulators in the TCGA melanoma cohort. **E** The gene mutation overview of twenty hub genes in the TCGA melanoma patients

In this study, the detailed flow chart for the prognostic predictive model construction is shown in Fig. 1. We collected twenty m6A regulators with obtainable expression data in TCGA and GTEx datasets. Compared with the normal skin tissues, nine out of twenty genes showed a significantly low expression level (*P* < 0.05) in 471 melanoma tissues, while the other eleven genes relatively represented a high expression (*P* < 0.05) (Fig. 2A–B).

As indicated in the PPI network, the m6A regulators exhibited intricate interactions among each other (Fig. 2C). YTHDF2 and RBM15 are the most relevant genes among these regulators. KIAA1429, METTL3, and METTL14 had the strongest correlation compared with the other seventeen regulators (Additional file 2: Figure S1). Furthermore, the correlation analysis based on the gene expressions displayed that part of the m6A regulators had the moderate to strong positive correlation compared with the other m6A genes (Fig. 2D). The alteration results of these m6A genes showed that VIRMA, IGF2BP1, IGF2BP3, ZC3H13, and YTHDF1 ranked as the most frequently altered genes. RBM15, VIRMA, IGF2BP3, and YTHDF3 were frequently overamplified in melanoma patients, while VIRMA displayed missense mutations with unknown significance (Fig. 2E).

### Construction of a three-gene risk signature with distinct prognostic value

**Fig. 3:**
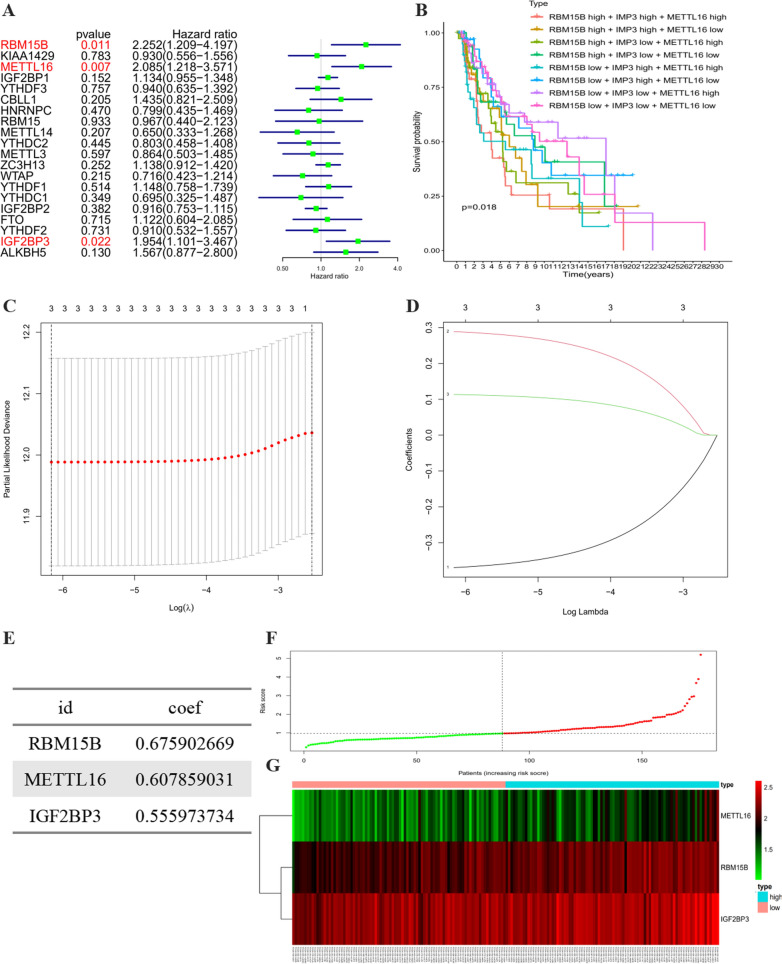
Construction of prognostic risk signature with 3 m6A RNA methylation regulators. **A** Univariate Cox analysis of m6A RNA methylation regulators in all TCGA melanoma cohort. **B** The combinations of low- or high- IGF2BP3, RBM15B, and METTL16 genes expression in the TCGA dataset. **C**–**D** LASSO Cox regression analysis of the selected three m6A RNA methylation regulators. **E** The coefficients estimated by multivariate Cox regression. **F** The distributions of risk scores. **G **The distributions of risk scores and OS status. The red dots represent high-risk patients, green dots represent low-risk patients

We included a detailed classification to summarize the distribution of demographic characteristics melanoma patients in Additional file 1: Table S1. Then the melanoma dates without complete survival information were excluded from our study. The entire group (n = 352) was randomly divided into a training subgroup (n = 176) (Additional file 1: Table S2) and a testing subgroup (n = 176) (Additional file 1: Table S3) by using the “caret” R package. There is no significant difference of the distribution of clinical features between the two subgroups (Additional file 1: Table S4). To investigate the prognostic value of m6A regulators in melanoma, univariate Cox regression analysis was exerted in the training cohort to identify hub regulators associated with OS. The results showed that three (RBM15B, METTL16, and IGF2BP3) out of twenty regulators were notably associated with OS (Fig. 3A, p < 0.05). The combinations of low- or high- IGF2BP3, RBM15B, and METTL16 genes expression in the TCGA dataset were also assessed. Patients with low expression of these three genes showed a greater survival advantage (Fig. 3B). Then, the LASSO Cox regression analysis was applied to better predict the clinical outcomes of melanoma. Based on the minimum criteria, the three genes were screened out (Fig. 3C and D). Next, the three hub genes were subjected to a step-by-step multivariate Cox regression for constructing the perfect risk signature (Fig. 3E and Table 1). Coefficients obtained from multivariate Cox analysis were conducted to calculate each melanoma patient’s risk score utilizing the following formula: risk score = (0.675902669) × RBM15B + (0.6078590311) × METTL16 + (0.555973734) × IGF2BP3. The distributions of the three-gene signature-based risk score and survival time were presented in Fig. 3F and G.

**Table 1:** The coefficients estimated by multivariate Cox regression

Id	coef	HR	HR.95L	HR.95H	*P* value
RBM15B	0.675902669	1.965806647	1.022577166	3.779074973	0.042670999
METTL16	0.607859031	1.836495307	1.067106328	3.160617575	0.028203545
IGF2BP3	0.555973734	1.743637998	1.005368082	3.024040175	0.047813655

### Validation of the prognostic role of the three-gene risk signature

**Fig. 4:**
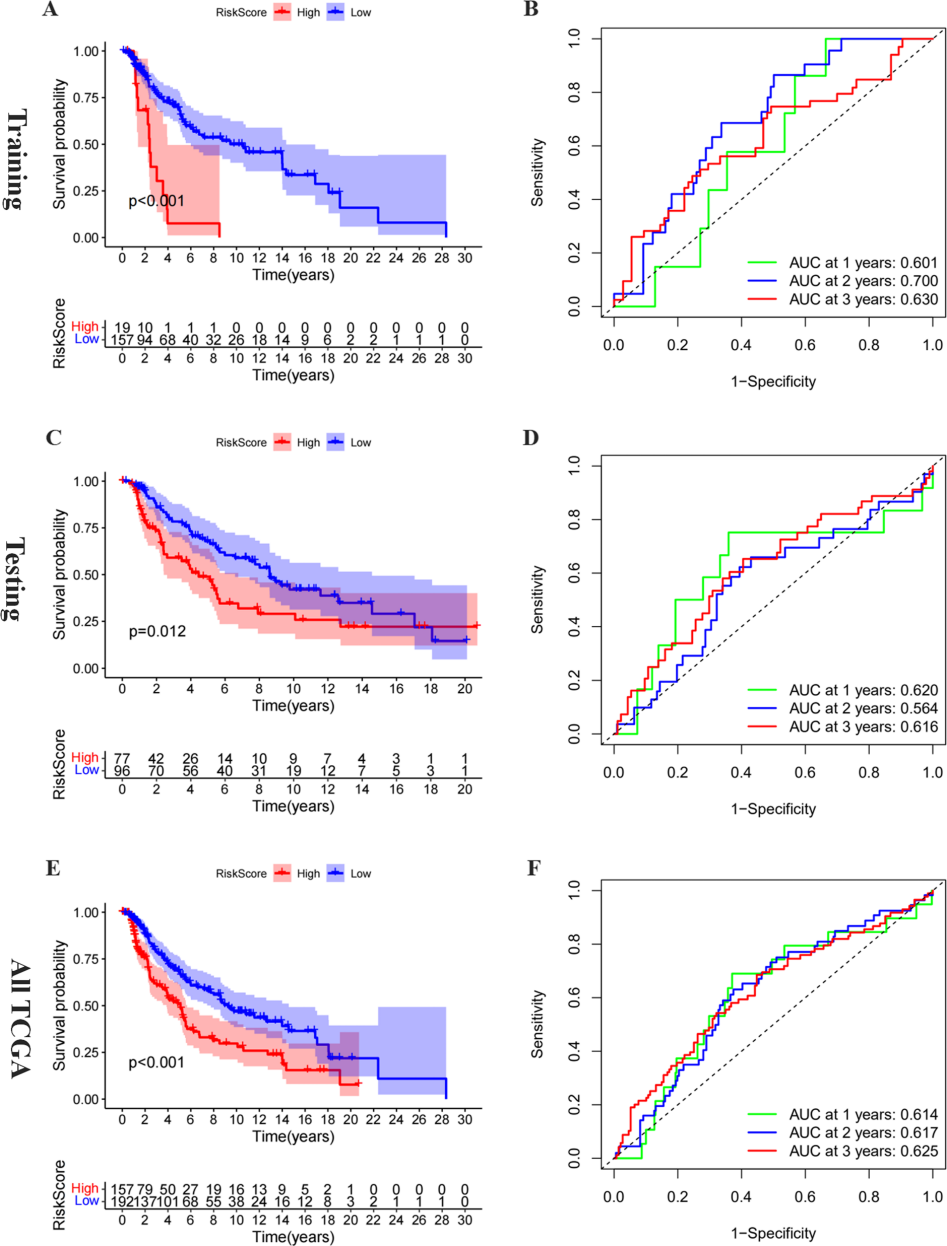
Validation of the prognostic risk signature. **A** The survival analysis of the three subgroups stratified based on the median of risk scores calculated by multivariate Cox result in the training group. **D** The ROC curve for evaluating the prediction efficiency of the prognostic signature in the training group. **B** The survival analysis of the two subgroups stratified based on the median of risk scores calculated by the prognostic risk signature in the testing group. **E** The time dependent ROC curve for evaluating the prediction efficiency of the prognostic signature in the testing group. **C** The survival analysis of the two subgroups stratified based on the median of risk scores calculated by the prognostic risk signature in the ALL TCGA group. **F** The time dependent ROC curve for evaluating the prediction efficiency of the prognostic signature in the ALL TCGA group

The melanoma patients in the training cohort were separated into the high- and low-risk groups based on the median risk score. The OS between these two groups was compared to evaluate the prognostic value of the three-gene risk signature. The results revealed that patients in the low-risk group had notably higher survival rates and times than those in the high-risk group (Fig. 4A, P < 0.05). The time-dependent ROC curve pointed out that the prognostic risk signature had an appropriate prediction efficiency with the AUC values equal to 0.601, 0.700, and 0.630 of 1, 2, and 3 years (Fig. 4B). The Kaplan–Meier curve suggested that melanoma patients in the high-risk group had a worse OS (*P* < 0.05), based on the median value of risk score, compared to those with low risk in the test cohort and all TCGA cohort (Fig. 4C and E). The ROC curves demonstrated that risk score in the validation cohorts had stable predictive performances with AUC equal to 0.620 and 0.614 of 1 year, respectively (Fig. 4D and F). The distributions of the risk score, survival time, and expression profiles were shown in Additional file 3: Figure S2A–C. Overall tumor mutation burden (TMB) reflects the number of mutations in tumor cells and is a quantifiable biomarker [24]. We have calculated the TMB of the melanoma genome and explored the correlation between risk score and TMB (Additional file 3: Figure S2D). There was no correlation between risk score and TMB in our research. Meanwhile, we also explored the relationship between the signature and melanoma metastasis in all TCGA and subgroups (Additional file 1: Table S5). It seems that the signature was not significantly related to metastasis. In all, these results demonstrated that this three-gene prognostic signature could effectively screen out high-risk melanoma patients corresponding to poor clinical outcomes.

### The signature-based risk score was an independent prognostic factor in melanoma

**Fig. 5:**
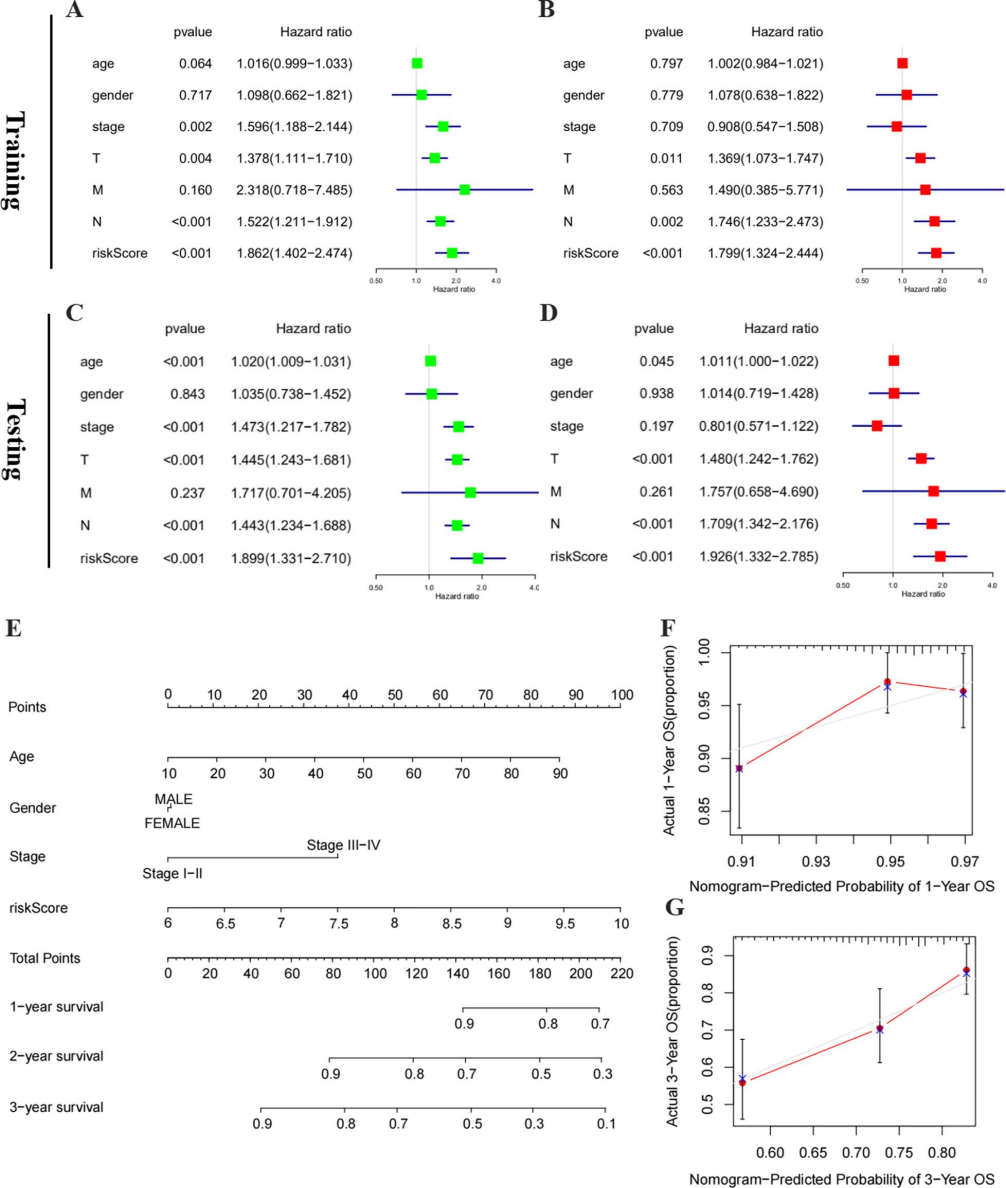
Identification of the independent prognostic factors in the training and validation group. **A** Univariate Cox analyses of the signature-based risk score and clinicopathological parameters in the training group. **B** Multivariate Cox analyses of the signature-based risk score and clinicopathological parameters in the training group. **C** Univariate Cox analyses of the signature-based risk score and clinicopathological parameters in the validation group. **D** Multivariate Cox analyses of the signature-based risk score and clinicopathological parameters in the validation group. **E** Prognostic nomogram for melanoma patients. **F–G** Calibration curves for the nomogram at 1-, and 3-year

To further explore whether the risk score could be served as an independent prognostic factor, analyses of univariate and multivariate Cox regression were performed in the training group. The univariate Cox analysis demonstrated that signature-based risk score was obviously associated with worse OS (HR = 1.862, *P* < 0.001) (Fig. 5A). Then, all of the above variables were applied to the multivariate Cox analysis. Significantly, this risk score was still evaluated as an independent risk factor for worse OS (HR = 1.799, *P* < 0.001) of melanoma patients (Fig. 5B). Consistent with the training cohort, the univariate analysis revealed that the high-risk score (HR = 1.899, *P* < 0.001) was remarkably associated with a poor OS in the validation cohort (Fig. 5C). The multivariate analysis further reflected that signature-based risk score exerted as an independent prognostic indicator (HR = 1.926, *p* < 0.001) (Fig. 5D). Hence, in melanoma, these data revealed that the risk score based on risk signature might be an independent prognostic factor.

Next, based on variables of three hub genes (IGF2BP3, METTL16, and RBM15B) expression derived from the all TCGA cohort, a nomogram was constructed to predict the 1-, 2-, and 3-year survival probabilities of melanoma patients. Four independent prognostic parameters of OS, including m6A WERs risk scores, age, gender, and stage, were integrated into the nomogram (Fig. 5E). The calibration plots indicated excellent consistency between actual observations and the predicted 3- and 5-year OS rates in the all TCGA cohort (Fig. 5F–G).

### Evaluation of the prognostic value of the three-gene risk signature

Then, subgroup analysis was further conducted to evaluate the prognostic role of the three-gene risk signature in melanoma patients with available clinicopathological factors, including gender, age, and stage. In Additional file 4: Figure S3A–F, high-risk patients had dramatically poorer OS compared with patients with low-risk (*P* < 0.05) in the training cohort except for patients of the subgroup with age and male. These demonstrated that the hub risk genes possessed stable discrimination capacity for patients with dissatisfactory prognosis. Moreover, subgroup analysis in the testing cohort suggested consistent outcomes that high-risk patients had a poorer OS compared with the low-risk patients except for patients in stage I–II (*P* < *0.05*) (Additional file 5: Figure S4A–F). These results above convincingly assessed the prognostic value of this three-gene risk signature in melanoma patients.

ROC curve of OS was used to reveal the predictive performance of the three-gene risk signature and clinical covariates in the training group. The AUC value of risk score, age, gender, clinical-stage, tumor, metastasis, and node with clinical data were 0.699, 0.547, 0.447, 0.647, 0.643, 0.504, and 0.684 respectively in the training cohort (Additional file 6: Figure S5A). These results indicated that the risk signature had a better ability to predict the survival of melanoma patients than did clinical factors.

To compare of the m6A signature with other confirmed melanoma prognostic biomarkers, we performed ROC analyses of other biomarkers in the same way the 3-gene signature was analyzed. The melanoma prognostic predictors from other 3 studies were selected for comparison [25–27]. The ROC curve analyses were performed, and the area under the ROC curve (AUC) was measured. Our signature curves demonstrated the AUC values for 2-year and 3-year OS of the m6A risk signature were 0.700 and 0.630, respectively, which were higher than the values of 7-gene signature (2-year: AUC = 0.645, 3-year: AUC = 0.638), 9-gene signature (2-year: AUC = 0.582, 3-year: AUC = 0.610), and 10-gene signature (2-year: AUC = 0:595, 3-year: AUC = 0.572) (Additional file 6: Figure S5B–C). These results underscored that our m6A risk gene signature was a better predictor for the prognosis assessment of melanoma and provided stability, reliability, and veracity in predicting OS. Meanwhile, we compared the prediction effect of the 3-gene signature with that of the other models through decision curve analysis (DCA) curves (Additional file 6: Figure S5D–E), which showed good positive net benefits in the prognostic model among most of the threshold probabilities at different time points (death at 2 and 3 years). Moreover, −2 log likelihood (−2LL) value of the models indicated that the goodness of fit of our regression equation (− 2LL = 581.82) was better than the 7 gene model (− 2LL = 593.28), the 9 gene model (− 2LL = 593.98) and the 10 gene model (− 2LL = 596.56) (Additional file 1: Table S6).The results showed that the performance of our model was beneficial and was better than that of the other models.

### Potential therapeutic value of the three-gene risk signature

The TCGA data was utilized to explore potential carcinogenic mechanisms associated with risk-related differentially expresses genes by GO and KEGG term enrichment analysis (Additional file 7: Figure S6A–B). The functional and pathway annotations by GO term enrichment analysis demonstrate that the aberrantly expressed genes participate in melanoma-related biological processes, such as epidermis and skin development, keratinocyte differentiation, cornification, and establishment of skin barrier. For cellular component, these target genes are significantly enriched in the desmosome and cornified envelope. For molecular function, they are significantly enriched in serine-type endopeptidase activity and chemorepellent activity. In the KEGG term enrichment analysis, the functional and pathway annotations demonstrate that the aberrantly expressed genes participate in histidine metabolism.

**Fig. 6:**
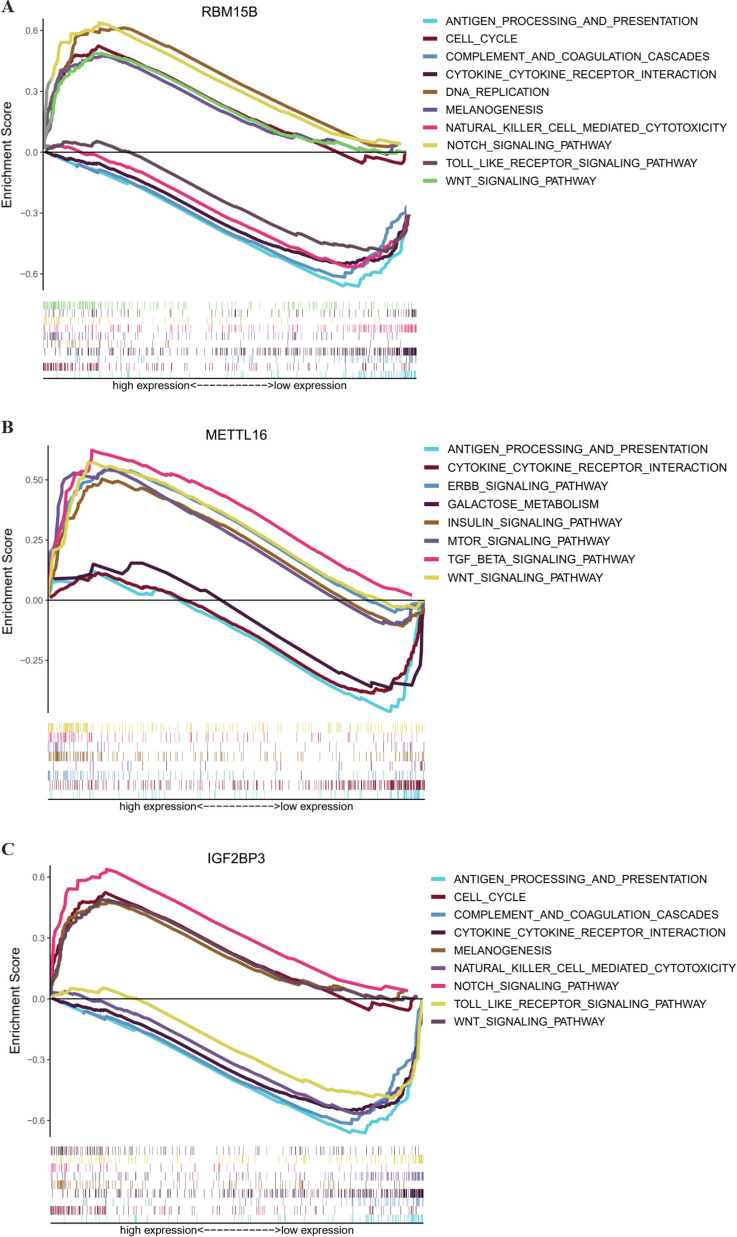

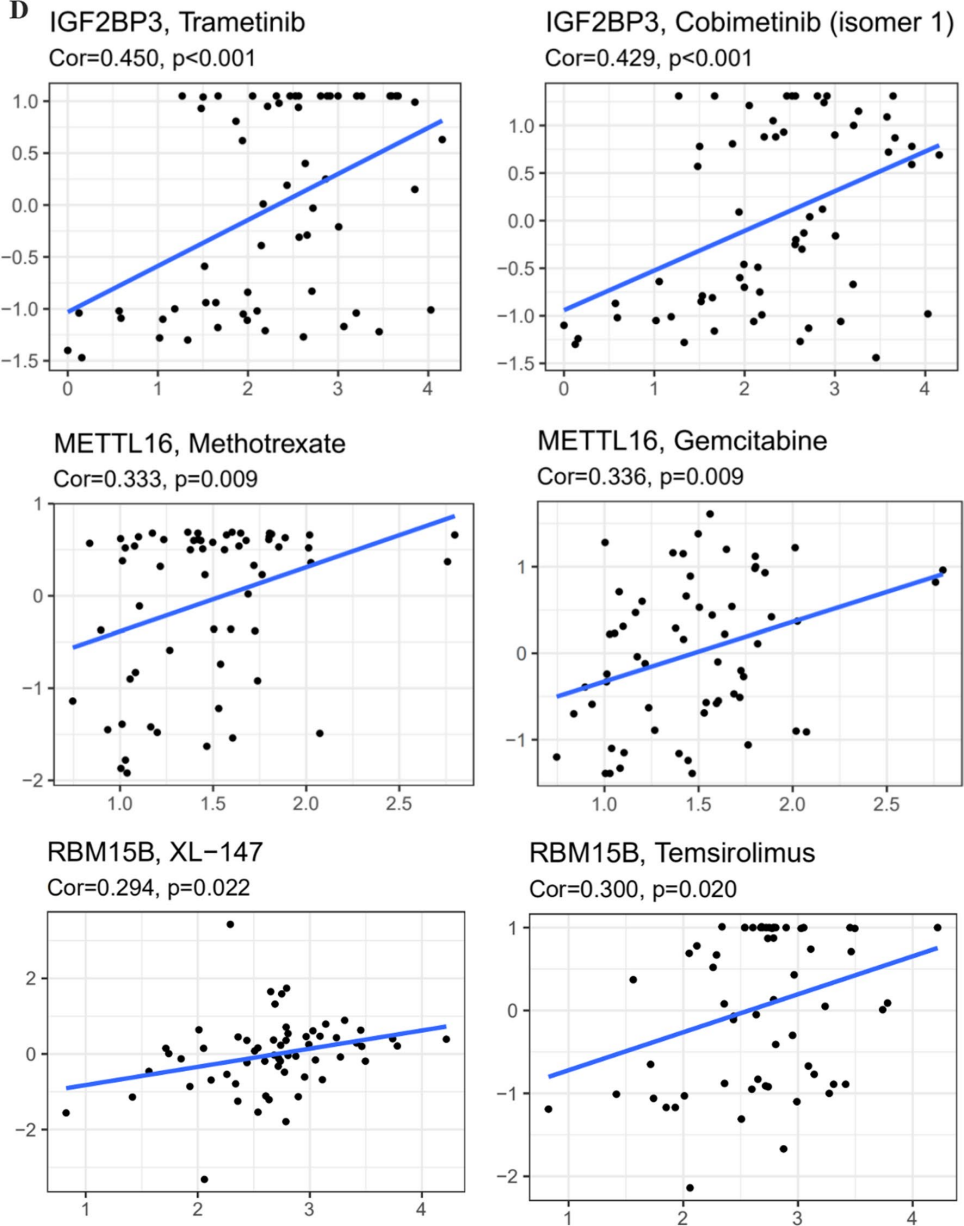
Functional annotation of hub gene in human cancers. **A**–**C** GSEA revealed the expression of IGF2BP3/ RBM15B/METTL16 was enriched for hallmarks of malignant tumors. **D** Trametinib and cobimetinib may exhibit sensitivity for melanoma with IGF2BP3 mutations, temsirolimus and XL-147 may exhibit sensitivity for melanoma with RBM15B mutations, gemcitabine and methotrexate may exhibit sensitivity for melanoma with METTL16 mutations (CellMiner)

Finally, the TCGA data was utilized to explore potential carcinogenic mechanisms with GSEA. IGF2BP3 might be involved in signal pathways including melanogenesis, notch signaling, wnt-signaling, cytokine–cytokine receptor interactions and so on (Fig. 6A). In addition, antigen processing and presentation, cell cycle, cytokine–cytokine receptor interactions, wnt-signaling, melanogenesis, and other cancer pathways were differentially enriched with high RBM15B expression of melanoma patients (Fig. 6B). While antigen processing, wnt-signaling, TGF beta signaling, mTOR signaling were differentially enriched with high METTL16 expression (Fig. 6C).

To explore the obtainable effects of anti-tumor drugs, CellMiner database was performed to identify sensitive and selective drugs for melanoma patients with or without IGF2BP3, RBM15B, and METTL16 mutations. The CellMiner screening results revealed that trametinib and cobimetinib exhibited sensitivity for melanoma patients with IGF2BP3 mutations. Temsirolimus and XL-147 exhibited sensitivity for melanoma with RBM15B mutations. In addition, gemcitabine and methotrexate exhibited sensitivity for melanoma with METTL16 mutations (Fig. 6D). The results revealed that IGF2BP3, RBM15B, and METTL16 mutations may be potential biomarkers for certain anti-tumor therapies.

### Validation of the expressions of the three hub genes in clinical samples

**Fig. 7:**
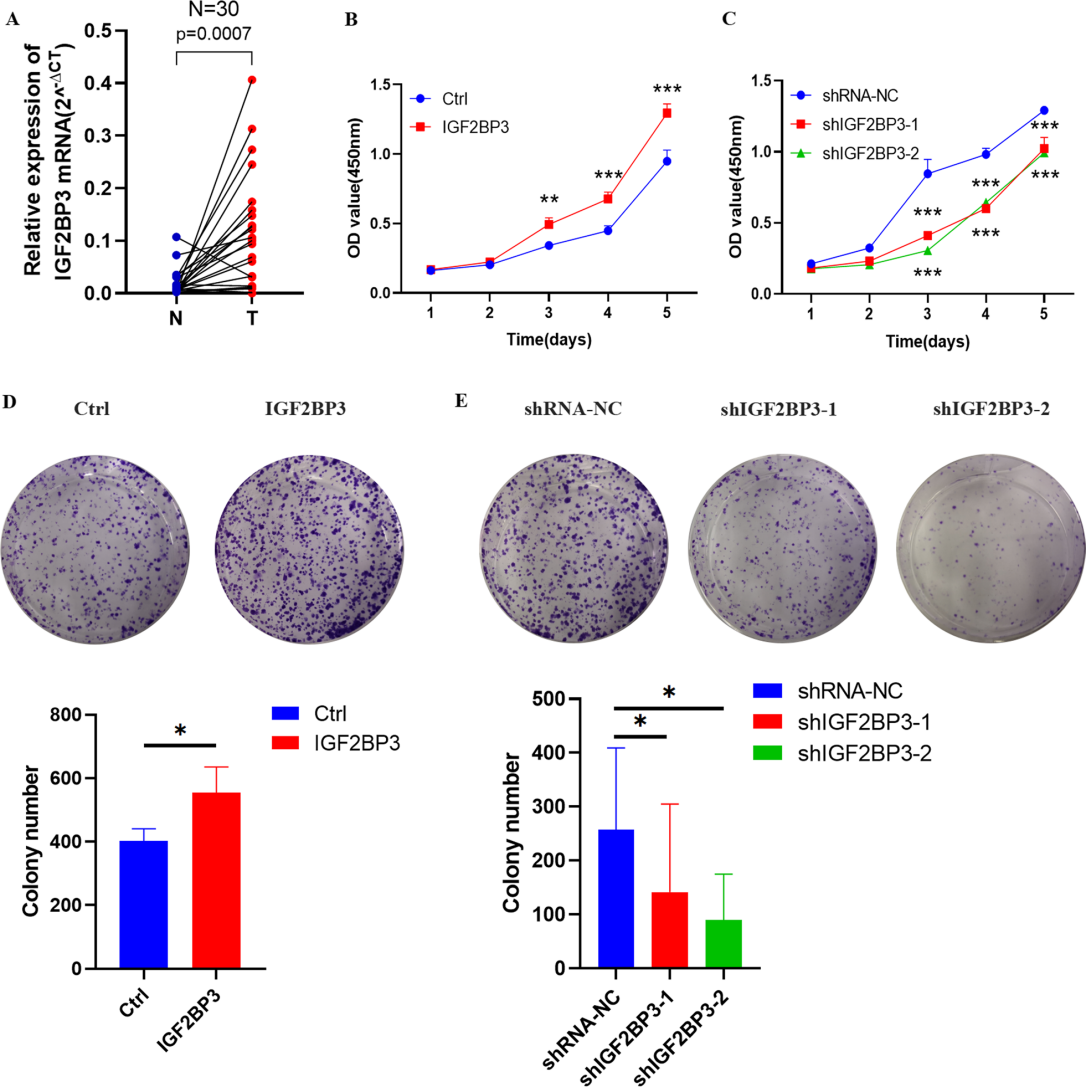

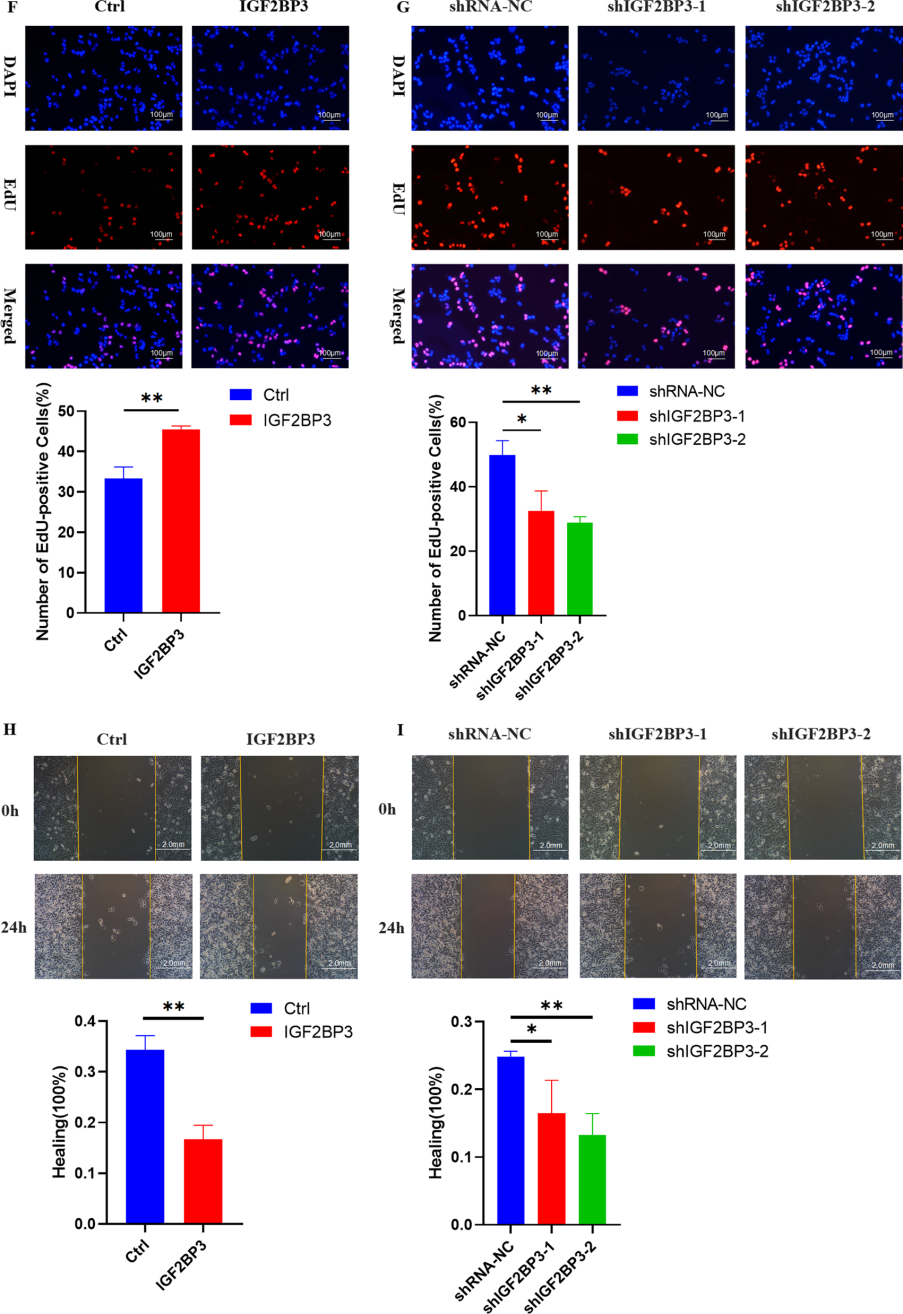
IGF2BP3 promoted melanoma cell proliferation and migration in vitro*.* **A** RT-qPCR analysis of IGF2BP3 expression of mRNA in 30 paired fresh melanoma tissues (T) and matched adjacent normal tissues (N) quantified after transfection. **B**–**C** Effects of IGF2BP3 overexpression and knockdown on cell proliferation by CCK-8 assays in A375 cells. (*P*-values were calculated using ANOVA test.). **D**–**E** Effects of IGF2BP3 overexpression and knockdown on cell proliferation by colony formation assays in A375 cells. **F**–**G** Effects of IGF2BP3 overexpression and knockdown on cell proliferation by EdU staining assay in A375 cells. (Scale bar, 100 μm). **H**–**I** Effects of IGF2BP3 overexpression and knockdown on cell migration by wound healing assay in A375 cells. (Scale bar, 2.0 mm). (Data are shown as the mean ± SD of three replicates. For statistical comparison between two independent experimental groups (Student’s t-test) and among more than two experimental groups (ANOVA test), appropriated statistical tests were assayed. *P < 0.05, **P < 0.01, ***P < 0.001.)

Immunohistochemical results analyzed from the HPA dataset showed that the three genes were overexpressed in melanoma tissues (Additional file 8: Figure S7A–C). Significantly, a consistent expression pattern of the three genes was validated in our clinical samples (tumor samples vs normal samples, n = 30). The results demonstrated that IGF2BP3 mRNA level was upregulated in melanoma tissues as well, while RBM15B and METTL16 was insignificant (Fig. 7A and Additional file 9: S8A), though high expression of RBM15B and METTL16 were associated with poor OS in TCGA database (Additional file 10: Figure S9A–B). Therefore, we further explored the expression and function of IGF2BP3 in melanoma. Correlations between the expression of IGF2BP3 and clinicopathological features in melanoma patients were calculated, IGF2BP3 level was significantly correlated with lymph node metastasis in melanoma (Additional file 1: Table S7). And IGF2BP3 showed a significantly higher expression level (P < 0.05) in metastasis melanoma tissues than in the primary melanoma tissues (Additional file 10: Figure S9C). The above results indicated that IGF2BP3 may contribute to melanoma progression.

### IGF2BP3 promoted the proliferation and migration in melanoma cells

Firstly, qRT-PCR and Western blot analysis revealed that IGF2BP3 was stably knockdown and overexpressed in A375 cells (Additional file 9: Figure S8B–E). Secondly, the effects of IGF2BP3 down-regulation and up-regulation on cell proliferation were further examined in A375 cells. CCK-8, colony formation, and EDU assays indicated that IGF2BP3 knockdown significantly inhibited melanoma cell proliferation, and IGF2BP3 overexpression promoted melanoma cell proliferation (Fig. 7B–G). In addition, cell migration was elevated by IGF2BP3 overexpression in A375 cells, and reduced by IGF2BP3 knockdown in A375 cells (Fig. 7H–I).

### IGF2BP3 influenced immune cell infiltration

**Fig. 8:**
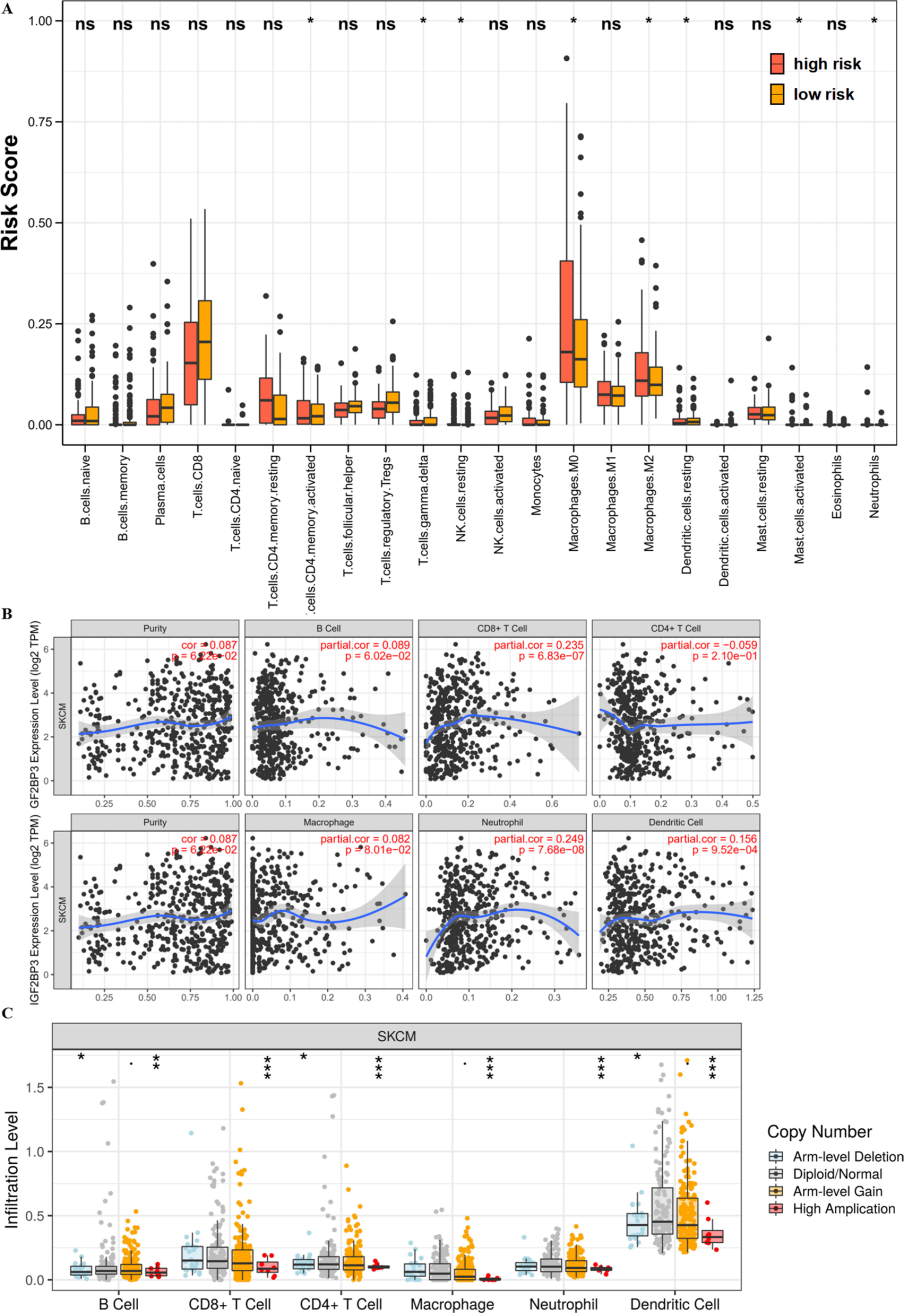
IGF2BP3 influenced immune cell infiltration. **A** The composition of the fractions of immune cells between high- and low-risk groups. **B** The correlation between IGF2BP3 expression and immune cell infiltration in the TIMER database. **C** The correlation between IGF2BP3 CNV and immune cell infiltration in the TIMER database

The abundance of 22 immune cell subtypes were estimated by the CIBERSORT method to explore the relevance of the risk score which derived from the three-gene risk signature with immune cell infiltration. Risk scores of resting dendritic cells and activated CD4 T memory cells were higher in the high-risk set compared with the risk scores in the low-risk set. M0 macrophages and M2 macrophages presented a higher fraction in the low-risk group compared with the high-risk group (Fig. 8A). The correlations of IGF2BP3 and immune cells is explored in melanoma using the Tumor Immune Estimation Resource (TIMER; cistrome.shinyapps.io/timer) database. And, the results displayed that IGF2BP3 was correlated with CD8 + T infiltration (r = 0.235, p = 6.83E−7), Neutrophil infiltration (r = 0.249, p = 7.68E−8) and Dendritic cell infiltration (r = 0.156, p = 9.52E−4) (Fig. 8B). Particularly, IGF2BP3 CNV has evidently correlated with immune infiltration in melanoma, including B cells, CD4 + T cells, CD8 + T cells, macrophages, neutrophils and dendritic cells (Fig. 8C). Whereas, IGF2BP3 expression was associated with various immune molecular markers involving M2 Macrophage, indicating its role in regulating tumor immunity (Table 2). These results indicated the potential association between IGF2BP3 and immune cell infiltration in tumor microenvironment of melanoma. As melanoma is correlated with higher level of inflammation, it’s relatively evident that each marker upregulated to tumor initiation and progression could be correlated to immune cell markers. Some further detailed experiments or clinic studies are needed for proving this speculation.

**Table 2:** Correlation analysis between IGF2BP3 and markers of immune cells in TIMER

Description	Gene markers	SKCM			
		None		Purity	
		Cor	P	Cor	P
CD8 + T cell	CD8A	− 0.012	0.794	− 0.016	0.738
	CD8B	− 0.022	0.636	− 0.028	0.553
B cell	CD19	− 0.005	0.907	− 0.011	0.810
	CD79A	− 0.084	0.068	− 0.086	0.066
T cell (general)	CD3D	− 0.048	0.302	− 0.053	0.254
	CD3E	− 0.065	0.158	− 0.069	0.140
	CD2	− 0.029	0.523	− 0.035	0.456
Monocyte	CD86	0.094	**0.042**	0.094	**0.044**
	CD14	0.028	0.547	0.038	0.421
	CD115(CSF1R)	0.065	0.162	0.070	0.133
TAM	CCL2	0.063	0.171	0.068	0.146
	CD68	0.081	0.078	0.067	0.152
	IL10	0.165	** < 0.001**	0.167	** < 0.001**
M1 Macrophage	INOS(NOS2)	− 0.015	0.743	− 0.009	0.844
	IRF5	0.020	0.671	0.027	0.571
	COX2(PTGS2)	0.148	**0.001**	0.151	**0.001**
	CD40	0.019	0.676	0.025	0.594
M2 Macrophage	CD206(MRC1)	0.165	** < 0.001**	0.177	** < 0.001**
	CD163	0.180	** < 0.001**	0.189	** < 0.001**
	VSIG4	0.099	**0.031**	0.109	**0.020**
	CD200R1	0.147	**0.001**	0.146	**0.002**
	MS4A4A	0.130	**0.005**	0.135	**0.004**
Natural killer cell	KIR3DL1	− 0.055	0.230	− 0.063	0.175
	KIR3DL2	− 0.080	0.083	− 0.076	0.104
	KIR3DL3	− 0.122	**0.008**	− 0.125	**0.008**
	KIR2DS4	− 0.122	**0.008**	− 0.084	0.071

Bold values represent significantly different (*P* < 0.05)

## Discussion

Owing to the heterogeneity of melanoma, it is demanding to use existing therapeutic strategies to treat different melanoma patients in decades. Although most past studies tendentiously focused on using human cell lines, tissues, or animal models to intervene at the level of a single gene or protein, several potent computational models have been constructed to assess disease-related mRNAs [28, 29]. An improved understanding of the specific types of gene expression profiles would assist in advancing the most specialized and individual therapeutic methods for different patients and effectively predict patients’ clinical outcomes.

The dysregulation of m6A regulatory protein evokes downstream RNA metabolism disorders and participates in the progression of various tumors [30–34]. The diverse functions of m6A genes involved in different tumor types indicated that the regulation of m6A genes methylation modification levels is extremely complex. While in melanoma, m6A methylation modification also played a dual role. For instance, the ‘writers’ METTL3 is upregulated and governs in invasion/migration through MMP2 in melanoma [35]. As for the ‘erasers’, ALKBH5 promotes the progression of uveal melanoma [36] and sensitizes tumors to cancer immunotherapy in melanoma [37]. Also, knockdown of FTO markedly improves cell sensitivity to interferon (IFN)-γ and anti-PD-1 therapeutics in melanoma, which suggested a promising anticarcinogenic therapy [38]. The ‘readers’, such as YTHDF1, could inhibit ocular melanoma by mediating m6A modification of HINT2 mRNA [39]. These findings provide a framework for the development and usage of m6A WREs inhibitors in melanoma treatment. However, these studies focused on the m6A WREs at the level of a single gene, whereas the combination of m6A WREs with clinicopathological parameters has shown great potential in the prognosis prediction of cancers, such as hepatocellular carcinoma, lung squamous cell carcinoma, and renal papillary cell carcinoma [40–42].

Several researches showed that the prognostic m6A gene signature contributes to the personalized prediction of cancer prognosis and acts as a potential biomarker which reflecting patients’ response to therapies in glioblastoma, pancreatic cancer, and colorectal cancer [15, 16, 43]. First, we constructed the prognostic risk signature among m6A regulators, this can potentially help prognosticate melanoma. Second, prognostic risk signature could act as independent prognostic markers and convincing clinicopathological parameter predictors. Third, the three prognostic panel genes (IGF2BP3, METTL16, and RBM15B) not only influenced the prognosis and clinicopathological features but were also closely correlated with tumorigenesis key signaling pathways, and hallmarks of malignant melanoma. In recent studies, the three genes were reported to play an oncogenic role in cancers respectively [44, 45]. We further verified the three key genes by qRT-PCR in our clinical samples, revealing that IGF2BP3 was upregulated in melanoma tumor tissues. Moreover, the expression level of IGF2BP3 was significantly related to the N stage and lymph node metastasis, suggesting that IGF2BP3 was intimately related to the malignancy progression and clinical features. Besides, RBM15B and METTL16 in the signature may influence melanoma tumor promotion because they were associated with poor OS in the TCGA database. However, there are no significant differences in the expression level of RBM15B and METTL16 between normal and tumor samples in our clinical simple so we did not study the role of RBM15B and METTL16 further. It may be because our sample size is small, so the follow-up significance of RBM15B and METTL16 needs to be further explored.

Several evidences have displayed the involvement of m6A regulators modification in innate and adaptive immune responses [42, 46], but its mechanism of shaping the tumor microenvironment in melanoma remains poorly understood. We have verified the oncogenic functions of IGF2BP3 in melanoma via cell functional experiments. Meanwhile, GSEA analysis showed that IGF2BP3 was also primarily related to cytokine–cytokine receptor interactions, antigen processing and presentation, and natural killer cell mediated cytotoxicity, wnt signaling and mTOR signaling pathways. Moreover, it was reported that IGF2BP3 could facilitate tumor immune escape by down-regulating the stress-induced ligands MICB and ULPB2 in colorectal carcinoma [47]. These results suggested that IGF2BP3 may influence via immune cells or immune-associated pathways. Remarkably, immune infiltration is an important factor in the tumor microenvironment and immunotherapy plays vital role in the development and prognosis of melanoma [26, 48, 49]. Recent studies also focused on the immune-related prognostic signature associated with immune infiltration in melanoma. For instance, Luo et al. displayed uveal melanoma patients' prognosis had close interactions with the immunodominant tumor environment [50]. Various immune cells contributes to the tumor progression of melanoma including adaptive immune CD8+ T-lymphocytes, macrophages, and so on [51]. Then we found that risk score was positively involved in the level of infiltration by various immune cells (CD4+ T cells, dendritic cells and so on). Meanwhile, the expression of IGF2BP3 was positively associated with the infiltration level of various immune cells (CD8+ T cells, neutrophils, dendritic cells and so on) and was significantly correlated with various immune molecular markers of immune cells. In anti-CTLA4-treated patients of melanoma, several studies showed that a reduction in the frequency of naive-phenotype CD4+ or CD8+ T cells were associated with better OS in the blood [52]. Intra-tumoral injection of dendritic cells has been proven to enhance anti-tumor immunity of melanoma-bearing mice and patients with advanced melanoma [53]. These discoveries imply that risk score and IGF2BP3 might be involved in immune infiltration governing in melanoma.

In our study, IGF2BP3 in melanoma has been linked with tumourigenic properties and poor prognosis, making it a potential target for drug development. Previous studies showed BRAF inhibitors (e.g. vemurafenib and dabrafenib) and MEK inhibitors (e.g. trametinib and cobimetinib) have been shown to be efficient in providing rapid tumor response, prolonging progression-free survival, and bettering OS in BRAF V600-mutated melanoma [54]. In our study, cobimetinib and trametinib exhibited sensitivity for melanoma with IGF2BP3 mutations. Also, methotrexate could sensitize drug-resistant metastatic melanoma cells to BRAF V600E inhibitors dabrafenib and encorafenib [55, 56]. Combining with temsirolimus, the treatment of BRAF V600E-mutated melanoma brain metastases cell lines may be effective in vitro [57]. In our study, methotrexate and temsirolimus exhibited sensitivity with METTL16 and RBM15B mutations respectively. These results implicated that IGF2BP3, METTL16, and RBM15B expressions might be associated with drug responses in melanoma that warrant further study. Further, this prognostic scoring system might provide an accurate method to help both clinicians and patients perform survival evaluations and select individualized treatment options.

IGF2BP3 has indeed been reported to participate in tumorigenicity in numerous kinds of cancers included melanoma. Therefore, we thought IGF2BP3 was a gene worthy for further analysis of its strong correlation with melanoma. This research provided new insights into the epigenetic comprehension and treatment measures of m6A regulators in melanoma. The novelty of the paper lies in the following aspects. Firstly, compared with previous bioinformatics studies that only constructed a prognostic model [49, 58, 59], we validated our results with our clinical samples and cell experiments. Besides, we compared our model with other existing published signatures and found that our m6A risk gene signature was a better predictor for the prognosis assessment of melanoma. Secondly, we explored the relevance of the signature and IGF2BP3 with immune infiltration in melanoma, which highlighted the relationship between tumor epigenetic heterogeneity and immune contexture. Meanwhile, we explored the obtainable effects of anti-tumor drugs for melanoma patients with IGF2BP3 mutations, revealing significantly epigenetic regulators of melanoma and novel approaches in precise and personalized medicine therapy. Thirdly, we also preliminary explored novel potential carcinogenic mechanisms of IGF2BP3 with GSEA as well as the role of RBM15B and METTL16 in melanoma, making our study more innovative and colorful. Overall, we performed a systematic evaluation of the underlying regulatory mechanisms of IGF2BP3 and its effects on prognosis, the infiltration of immune cells, the levels of CNV, and therapeutic sensitivity in melanoma.

However, there were several limitations of the present study. Our study was based on an individual source from TCGA, without external validation from other independent cohorts. Moreover, the in vivo studies and the accurate molecular mechanism in melanoma progression are needed for further clarification of these findings.

## Conclusion

Collectively, we identified a signature of three hub m6A genes to predict the OS of melanoma patients. Among these hub genes, IGF2BP3 might well have clinical value as diagnostic markers. Furthermore, this research was firstly analyzed with m6A genes in clinical samples that matched clinical pathological features and involved in immune infiltration conceiving the potential therapeutic target on BRAF and MEK in melanoma. These findings have improved our understanding of m6A RNA methylation and may provide useful guidance for their clinical use in melanoma.

## Supplementary Information

**Additional file 1: Table S1.** Clinical information of each melanoma patient in TCGA database. **Table S2.** Patients information in the training group. **Table S3.** Patients information in the testing group. **Table S4.** Clinicopathological characteristics of melanoma patients from the training and testing group. **Table S5.** Correlations between the signature and metastasis in TCGA melanoma patients. **Table S6.** The -2 log likelihood of our three-gene signature compared with the other three prognostic models. **Table S7.** Correlations between the expression of IGF2BP3 and clinicopathological features in melanoma patients (n = 30).

**Additional file 2: Figure S1.** RanK of hub genes in selected twenty RNA methylation regulators.

**Additional file 3: Figure S2.** The distributions of prognostic signature-based risk scores. (A) The distributions of prognostic signature-based risk scores and their corresponding expression profiles. (B) The distributions of risk scores. (C) The distributions of risk scores and OS status. The red dots represent high-risk patients, green dots represent low-risk patients. There was no correlation between risk score and TMB in the TCGA database.

**Additional file 4: Figure S3.** The survival analyses for the low-risk and high-risk subgroups stratified by clinicopathological parameters in the training group. (A–B) The survival analyses for the low-risk and high-risk subgroups stratified by age in the training group. (C–D) The survival analyses for the low- and high-risk subgroups stratified by gender in the training group. (E–F) The survival analyses for the low- and high-risk subgroups stratified by stage in the training group.

**Additional file 5: Figure S4.** The survival analyses for the low-risk and high-risk subgroups stratified by clinicopathological parameters in the testing group. (A–B) The survival analyses for the low-risk and high-risk subgroups stratified by age in the testing group. (C–D) The survival analyses for the low- and high-risk subgroups stratified by gender in the testing group. (E–F) The survival analyses for the low- and high-risk subgroups stratified by stage in the testing group.

**Additional file 6: Figure S5**. Validation of the m6A gene signature. (A) The AUC value of risk score, age, gender, clinical stage, tumor, metastasis, and node with clinical data in the training cohort. (B–C) The AUC value for 2-year and 3-year ROC of our m6A gene signature compared with other three gene-associated signatures. (D–E) The DCA curves for our m6A gene signature and other two gene-associated signatures in 2-year and 3-year.

**Additional file 7: Figure S6.** GO and KEGG pathway analysis of risk-related differentially expresses genes. (A) Bubble plots of biological process GO terms for risk-related differentially expresses genes. (B) Circle plots of biological process KEGG pathway analysis for risk-related differentially expresses genes.

**Additional file 8: Figure S7.** Protein expression of three hub genes detecting by an immunohistochemical assay in melanoma based on Human Protein Atlas website (www.proteinatlas.org). (A) Immunohistochemical staining showed the images of the protein expression of IGF2BP3 in melanoma. (B) Immunohistochemical staining showed the images of the protein expression of METTL16 in melanoma. (C) Immunohistochemical staining showed the images of the protein expression of RBM15B in melanoma.

**Additional file 9: Figure S8.** Construction of IGF2BP3 overexpressed cell lines. (A-B) RT-qPCR analysis of RBM15B and METTL16 expressions of mRNA in 30 paired fresh melanoma tissues (T) and matched adjacent normal tissues (N) quantified after transfection. (C-D) Western blot were used to confirm IGF2BP3 overexpression after transfection with lentivirus in A375 cell line. (Data are shown as the mean ± SD of three replicates. *P < 0.05, **P < 0.01, *** P < 0.001 by Student’s t-test.)

**Additional file 10: Figure S9.** The expression levels of the risk genes in the TCGA metastasis database. (A–B) High expression of RBM15B and METTL16 were associated with poor OS in TCGA database. (C) The expression level of IGF2BP3 was verified in 1 normal tissue, 104 primary tissues, and 368 metastasis tissues from TCGA database (UALCAN).

## Data Availability

All data generated or analyzed during this study are included either in this article or in the Additional files. The raw data supporting the conclusions of this manuscript will be made available by the authors, without undue reservation, to any qualified researcher.

## Abbreviations

ALKBH5: AlkB homolog 5
AUC: Area under the curve
CBLL1: Cbl proto-oncogene-like 1
DAVID: Database for Annotation, Visualization, and Integrated Discovery
DFS: Disease-free survival
FTO: Fat mass and obesity-associated protein
GO: Gene ontology
GTEx: Genotype-Tissue Expression
HNRNPC: Heterogeneous nuclear ribonucleoprotein C
IGF2BP1/2/3: Insulin-like growth factor 2 mRNA-binding protein 1/2/3
KEGG: Kyoto Encyclopedia of Genes and Genomes
LASSO: Least absolute shrinkage and selection operator
METTL3/14/16: Methyltransferase like 3/14/16
m6A: N6-methyladenosine
OS: Overall survival
RBM15: Binding motif protein 15
RBM15B: RNA binding motif protein 15B;
ROC: Receiver operating characteristic
TCGA: The Cancer Genome Atlas
WTAP: Wilms tumor 1-associated protein
YTHDC1/2/3: YTH domain-containing 1/2/3
YTHDF1/2: YTH N6-methyladenosine RNA-binding protein ½
ZC3H13: Zinc finger CCCH domain-containing protein 13
VIRMA: Vir like m6A methyltransferase associated
