# Supplementary material for: Effects of RNA methylation N6-methyladenosine regulators on malignant progression and prognosis of melanoma
Source: Cancer Cell Int. 2021 Aug 26;21:453. doi: 10.1186/s12935-021-02163-9 (PMC8393813; doi:10.1186/s12935-021-02163-9)
Supplement: Supplementary file 1 — Additional file 1: Table S1. Clinical information of each melanoma patient in TCGA database. Table S2. Patients information in the training group. Table S3. Patients information in the testing group. Table S4. Clinicopathological characteristics of melanoma patients from the training and testing group. Table S5. Correlations between the signature and metastasis in TCGA melanoma patients. Table S6. The -2 log likelihood of our three-gene signature compared with the other three prognostic models. Table S7. Correlations between the expression of IGF2BP3 and clinicopathological features in melanoma patients (n = 30). [file 12935_2021_2163_MOESM1_ESM.docx]

**Table S1 Clinical information of each melanoma patient in TCGA database.**

| **Variables** | **Group** | **Alive**  **(n = 252)** | **Dead**  **(n = 208)** | **Total**  **(n = 460)** |
| --- | --- | --- | --- | --- |
| Age  Gender  Stage  Clinical_T  Clinical_N  Clinical_M | ≤60  >60  Female  Male  <stage III  ≥stage III  unknow  TX  <T3  ≥T3  unknow  NX  N0  ≥N1  unknow  M0  M1 | 134 (53.2%)  118 (46.8%)  107 (42.5%)  145 (57.5%)  130 (51.6%)  122 (48.4%)  12 (4.8%)  24 (9.5%)  84 (33.3%)  132 (52.4%)  10 (4.0%)  21 (8.3%)  124 (49.2%)  97 (38.5%)  15 (6.0%)  223 (88.5%)  14 (5.6%) | 115 (55.3%)  93 (44.7%)  68 (32.7%)  140 (67.3%)  102 (49.0%)  106 (51.0%)  15 (7.2%)  20 (9.6%)  64 (30.8%)  109 (52.4%)  9 (4.3%)  14 (6.7%)  105 (50.5%)  80 (38.5%)  11 (5.3%)  188 (90.4%)  9 (4.3%) | 249 (54.1%)  211 (45.9%)  175 (38.0%)  285 (62.0%)  232 (50.4%)  228 (49.6%)  27 (5.9%)  44 (9.6%)  148 (32.2%)  241 (52.4%)  19 (4.1%)  35 (7.6%)  229 (49.8%)  177 (38.5%)  26 (5.7%)  411 (89.3%)  23 (5.0%) |

| **Table S2 Patients information in the train group.** | | |
| --- | --- | --- |
| **id** | **survival time (day)** | **survival status** |
| TCGA-D3-A51E | 5318 | 0 |
| TCGA-D3-A3CB | 5065 | 0 |
| TCGA-XV-A9W5 | 392 | 0 |
| TCGA-D3-A8GR | 3943 | 1 |
| TCGA-D3-A2JB | 5110 | 1 |
| TCGA-ER-A2NE | 613 | 1 |
| TCGA-EE-A29W | 5932 | 0 |
| TCGA-EE-A183 | 818 | 1 |
| TCGA-FR-A729 | 6716 | 0 |
| TCGA-EE-A2MF | 8174 | 1 |
| TCGA-EE-A2GK | 1665 | 0 |
| TCGA-EE-A2MM | 5107 | 1 |
| TCGA-W3-AA21 | 3195 | 1 |
| TCGA-EE-A3J7 | 1949 | 0 |
| TCGA-EE-A2GE | 4898 | 0 |
| TCGA-EE-A2M5 | 659 | 1 |
| TCGA-EE-A20H | 5118 | 1 |
| TCGA-EE-A2MN | 1446 | 1 |
| TCGA-EE-A2M6 | 3932 | 0 |
| TCGA-FS-A1Z4 | 854 | 1 |
| TCGA-W3-A824 | 6940 | 0 |
| TCGA-XV-A9W2 | 417 | 0 |
| TCGA-FS-A1Z0 | 6164 | 1 |
| TCGA-Z2-AA3V | 486 | 0 |
| TCGA-EE-A2GI | 1482 | 0 |
| TCGA-WE-AAA0 | 1065 | 0 |
| TCGA-EE-A17X | 907 | 1 |
| TCGA-D3-A8GI | 1780 | 1 |
| TCGA-EE-A2GJ | 2270 | 0 |
| TCGA-D3-A1Q7 | 4053 | 0 |
| TCGA-ER-A19D | 383 | 1 |
| TCGA-FR-A7UA | 1164 | 0 |
| TCGA-ER-A19M | 1857 | 1 |
| TCGA-EE-A3J3 | 5237 | 1 |
| TCGA-EE-A2A5 | 1195 | 1 |
| TCGA-EE-A3JA | 1618 | 1 |
| TCGA-EE-A2MT | 2166 | 0 |
| TCGA-EE-A2A1 | 3130 | 0 |
| TCGA-EE-A3JH | 3333 | 0 |
| TCGA-DA-A95X | 1513 | 0 |
| TCGA-D3-A5GL | 3826 | 0 |
| TCGA-XV-AAZW | 393 | 1 |
| TCGA-EE-A20B | 4070 | 0 |
| TCGA-EE-A181 | 1026 | 1 |
| TCGA-EE-A2ML | 6590 | 1 |
| TCGA-FS-A1ZP | 2273 | 1 |
| TCGA-D3-A5GO | 4195 | 0 |
| TCGA-EE-A3J4 | 3869 | 1 |
| TCGA-EE-A2GR | 1301 | 1 |
| TCGA-FS-A1YY | 6953 | 1 |
| TCGA-EB-A3XE | 180 | 0 |
| TCGA-D3-A51R | 1941 | 0 |
| TCGA-BF-A3DM | 601 | 0 |
| TCGA-EE-A2GN | 3106 | 1 |
| TCGA-EE-A2GD | 10346 | 1 |
| TCGA-EB-A85J | 360 | 0 |
| TCGA-FS-A4F0 | 2367 | 0 |
| TCGA-EE-A29Q | 2030 | 1 |
| TCGA-GN-A268 | 1910 | 1 |
| TCGA-BF-AAP2 | 405 | 0 |
| TCGA-D3-A2J6 | 1321 | 1 |
| TCGA-EE-A29B | 2588 | 1 |
| TCGA-D9-A4Z5 | 218 | 0 |
| TCGA-DA-A960 | 804 | 0 |
| TCGA-EE-A2GC | 2051 | 0 |
| TCGA-BF-A1PU | 387 | 0 |
| TCGA-FS-A4F2 | 1525 | 1 |
| TCGA-EB-A5UM | 414 | 0 |
| TCGA-EB-A3Y6 | 126 | 0 |
| TCGA-FR-A8YD | 1103 | 1 |
| TCGA-ER-A196 | 1785 | 0 |
| TCGA-EE-A29P | 1716 | 0 |
| TCGA-EB-A41B | 291 | 0 |
| TCGA-FS-A1ZF | 470 | 1 |
| TCGA-EB-A431 | 568 | 0 |
| TCGA-EB-A3HV | 39 | 0 |
| TCGA-EB-A1NK | 613 | 0 |
| TCGA-EB-A3XF | 278 | 0 |
| TCGA-BF-AAP4 | 335 | 0 |
| TCGA-IH-A3EA | 524 | 0 |
| TCGA-EB-A553 | 226 | 0 |
| TCGA-BF-A5EQ | 323 | 0 |
| TCGA-BF-A5ER | 327 | 0 |
| TCGA-BF-A5EO | 703 | 0 |
| TCGA-EB-A85I | 362 | 0 |
| TCGA-EB-A97M | 414 | 0 |
| TCGA-FR-A3R1 | 685 | 0 |
| TCGA-EB-A6QY | 382 | 0 |
| TCGA-BF-A9VF | 440 | 0 |
| TCGA-BF-A1Q0 | 831 | 0 |
| TCGA-BF-AAOX | 444 | 0 |
| TCGA-D3-A5GR | 5424 | 0 |
| TCGA-FS-A4FB | 813 | 1 |
| TCGA-D3-A2JC | 2639 | 0 |
| TCGA-EE-A2M8 | 601 | 1 |
| TCGA-D3-A3C7 | 1429 | 0 |
| TCGA-EB-A82B | 390 | 0 |
| TCGA-EB-A5SH | 1643 | 0 |
| TCGA-EE-A3JB | 6138 | 0 |
| TCGA-D3-A3CE | 1832 | 1 |
| TCGA-D3-A1QB | 2912 | 0 |
| TCGA-D3-A51J | 4414 | 0 |
| TCGA-EE-A3AB | 3733 | 0 |
| TCGA-D3-A3MR | 3151 | 0 |
| TCGA-DA-A1HY | 3580 | 0 |
| TCGA-EE-A3AD | 875 | 1 |
| TCGA-BF-AAP6 | 325 | 0 |
| TCGA-EB-A5KH | 619 | 1 |
| TCGA-ER-A3EV | 1429 | 1 |
| TCGA-FS-A1ZT | 1617 | 0 |
| TCGA-EE-A3J5 | 1124 | 1 |
| TCGA-D3-A2JG | 3453 | 1 |
| TCGA-GN-A26A | 988 | 1 |
| TCGA-EE-A2MQ | 1315 | 1 |
| TCGA-FS-A1ZN | 730 | 1 |
| TCGA-EE-A29G | 2192 | 1 |
| TCGA-D9-A6EC | 2015 | 0 |
| TCGA-EE-A3J8 | 1044 | 1 |
| TCGA-EE-A29A | 1927 | 1 |
| TCGA-3N-A9WD | 395 | 1 |
| TCGA-DA-A1HW | 428 | 0 |
| TCGA-D9-A3Z3 | 678 | 0 |
| TCGA-FS-A1ZA | 843 | 1 |
| TCGA-HR-A2OH | 2004 | 1 |
| TCGA-FR-A728 | 583 | 0 |
| TCGA-FW-A3TV | 411 | 0 |
| TCGA-EB-A4OY | 593 | 0 |
| TCGA-EB-A5VV | 214 | 0 |
| TCGA-ER-A197 | 424 | 1 |
| TCGA-BF-A3DL | 769 | 0 |
| TCGA-EE-A2GB | 1803 | 0 |
| TCGA-DA-A3F2 | 657 | 0 |
| TCGA-BF-A1PX | 282 | 1 |
| TCGA-EB-A57M | 399 | 0 |
| TCGA-EB-A5VU | 321 | 1 |
| TCGA-ER-A2NB | 857 | 1 |
| TCGA-DA-A1I7 | 1876 | 0 |
| TCGA-FS-A1ZW | 1505 | 0 |
| TCGA-EE-A17Y | 828 | 1 |
| TCGA-WE-A8JZ | 731 | 0 |
| TCGA-EB-A24D | 645 | 0 |
| TCGA-ER-A42K | 394 | 1 |
| TCGA-EB-A4OZ | 620 | 0 |
| TCGA-EE-A29R | 440 | 0 |
| TCGA-EE-A3AF | 420 | 1 |
| TCGA-D3-A3CF | 746 | 1 |
| TCGA-FR-A7U9 | 571 | 0 |
| TCGA-DA-A1IB | 454 | 0 |
| TCGA-D9-A4Z3 | 505 | 0 |
| TCGA-GF-A4EO | 591 | 0 |
| TCGA-EB-A551 | 590 | 0 |
| TCGA-D3-A1Q1 | 504 | 1 |
| TCGA-BF-A3DN | 717 | 0 |
| TCGA-EE-A182 | 447 | 1 |
| TCGA-WE-AAA3 | 600 | 0 |
| TCGA-ER-A199 | 279 | 1 |
| TCGA-D3-A2JK | 368 | 1 |
| TCGA-D3-A2JP | 1812 | 0 |
| TCGA-FS-A4FD | 2454 | 1 |
| TCGA-ER-A2NH | 1264 | 0 |
| TCGA-D9-A4Z2 | 190 | 1 |
| TCGA-D3-A51F | 1695 | 0 |
| TCGA-DA-A1I4 | 1093 | 1 |
| TCGA-D3-A2JD | 361 | 1 |
| TCGA-D9-A6EA | 766 | 0 |
| TCGA-EE-A2A2 | 1120 | 0 |
| TCGA-WE-A8K1 | 1492 | 0 |
| TCGA-EE-A29L | 79 | 1 |
| TCGA-FS-A4F9 | 1035 | 0 |
| TCGA-EE-A29D | 425 | 1 |
| TCGA-D3-A51N | 688 | 0 |
| TCGA-ER-A3PL | 1010 | 0 |
| TCGA-D3-A1Q8 | 854 | 1 |
| TCGA-ER-A19T | 270 | 1 |
| TCGA-D3-A5GS | 553 | 0 |
| TCGA-WE-A8K5 | 1860 | 1 |

Survival status: 0 represent alive, 1represent dead.

**Table S3 Patients information in the test group.**

| **id** | **survival time (day)** | **survival status** |
| --- | --- | --- |
| TCGA-EE-A20C | 4601 | 1 |
| TCGA-D3-A5GN | 4129 | 0 |
| TCGA-EE-A2MP | 7563 | 0 |
| TCGA-FS-A1YX | 1478 | 1 |
| TCGA-FS-A1ZJ | 1441 | 1 |
| TCGA-EE-A2MG | 3139 | 1 |
| TCGA-EE-A2GH | 6328 | 0 |
| TCGA-DA-A3F5 | 6446 | 0 |
| TCGA-EE-A3JI | 4648 | 1 |
| TCGA-EE-A2ME | 3141 | 1 |
| TCGA-D3-A8GS | 3564 | 1 |
| TCGA-FS-A4F8 | 5318 | 1 |
| TCGA-EE-A20F | 2785 | 0 |
| TCGA-FS-A1ZS | 4526 | 0 |
| TCGA-EE-A2MR | 4088 | 0 |
| TCGA-EE-A2MC | 1871 | 1 |
| TCGA-EE-A29H | 1966 | 0 |
| TCGA-EE-A2GU | 2884 | 0 |
| TCGA-FR-A8YE | 3176 | 0 |
| TCGA-EE-A2A6 | 2620 | 0 |
| TCGA-Z2-AA3S | 2950 | 0 |
| TCGA-EE-A2MU | 1620 | 0 |
| TCGA-D3-A2JF | 1888 | 0 |
| TCGA-EE-A2GS | 2470 | 1 |
| TCGA-EE-A29M | 1729 | 0 |
| TCGA-ER-A19E | 396 | 1 |
| TCGA-D3-A3C6 | 1766 | 1 |
| TCGA-EE-A29X | 545 | 1 |
| TCGA-FS-A4F5 | 874 | 1 |
| TCGA-EE-A29C | 2402 | 1 |
| TCGA-D3-A5GU | 3808 | 0 |
| TCGA-FR-A3YN | 2828 | 0 |
| TCGA-D3-A2J8 | 1992 | 1 |
| TCGA-ER-A2NC | 1333 | 1 |
| TCGA-FS-A1YW | 6598 | 1 |
| TCGA-D3-A1QA | 2765 | 0 |
| TCGA-D3-A2JH | 1280 | 0 |
| TCGA-EE-A184 | 2073 | 1 |
| TCGA-FR-A44A | 5299 | 0 |
| TCGA-XV-AAZV | 412 | 0 |
| TCGA-W3-A825 | 1917 | 1 |
| TCGA-EE-A2GO | 3857 | 0 |
| TCGA-D3-A8GJ | 7342 | 0 |
| TCGA-EE-A3AH | 4222 | 1 |
| TCGA-FS-A1ZR | 347 | 1 |
| TCGA-ER-A42L | 4533 | 0 |
| TCGA-EE-A2MD | 1438 | 1 |
| TCGA-FS-A1ZB | 1486 | 1 |
| TCGA-W3-AA1V | 1280 | 1 |
| TCGA-FS-A4F4 | 2028 | 1 |
| TCGA-W3-AA1W | 6666 | 0 |
| TCGA-D3-A8GQ | 884 | 1 |
| TCGA-EE-A2M7 | 877 | 1 |
| TCGA-W3-A828 | 3683 | 1 |
| TCGA-FS-A1ZK | 728 | 1 |
| TCGA-W3-AA1R | 3379 | 1 |
| TCGA-EE-A2MS | 4942 | 0 |
| TCGA-EE-A2GL | 2423 | 0 |
| TCGA-FS-A4FC | 1655 | 1 |
| TCGA-EB-A6QZ | 352 | 1 |
| TCGA-EE-A2A0 | 1424 | 1 |
| TCGA-D3-A8GK | 5177 | 0 |
| TCGA-WE-A8ZQ | 1818 | 0 |
| TCGA-EB-A299 | 378 | 0 |
| TCGA-FS-A1ZD | 1628 | 1 |
| TCGA-EE-A2GT | 1365 | 0 |
| TCGA-EE-A29S | 1864 | 1 |
| TCGA-Z2-A8RT | 839 | 0 |
| TCGA-FS-A1ZZ | 822 | 1 |
| TCGA-YG-AA3P | 439 | 0 |
| TCGA-BF-A1PZ | 853 | 0 |
| TCGA-EE-A2MI | 6225 | 1 |
| TCGA-EE-A17Z | 263 | 1 |
| TCGA-ER-A193 | 955 | 1 |
| TCGA-D3-A3BZ | 3976 | 0 |
| TCGA-EB-A44O | 81 | 0 |
| TCGA-FS-A1ZY | 824 | 1 |
| TCGA-D3-A8GM | 3259 | 1 |
| TCGA-FR-A8YC | 1059 | 1 |
| TCGA-FR-A2OS | 368 | 1 |
| TCGA-EB-A44P | 376 | 0 |
| TCGA-EB-A6R0 | 467 | 0 |
| TCGA-DA-A1I8 | 1368 | 0 |
| TCGA-D3-A3CC | 2644 | 0 |
| TCGA-EB-A82C | 17 | 0 |
| TCGA-EE-A2GM | 2296 | 0 |
| TCGA-FS-A1ZU | 808 | 1 |
| TCGA-BF-AAOU | 476 | 0 |
| TCGA-EB-A42Y | 440 | 0 |
| TCGA-BF-A1PV | 14 | 0 |
| TCGA-BF-A5ES | 490 | 0 |
| TCGA-BF-AAP7 | 318 | 0 |
| TCGA-ER-A19K | 469 | 1 |
| TCGA-FS-A1ZE | 1413 | 1 |
| TCGA-GF-A2C7 | 21 | 0 |
| TCGA-BF-AAP8 | 447 | 0 |
| TCGA-EB-A44N | 205 | 1 |
| TCGA-D3-A1Q3 | 507 | 1 |
| TCGA-YG-AA3N | 306 | 0 |
| TCGA-DA-A95Y | 430 | 1 |
| TCGA-EB-A3XC | 650 | 0 |
| TCGA-EB-A4P0 | 326 | 1 |
| TCGA-BF-AAP1 | 409 | 0 |
| TCGA-EE-A2MK | 4825 | 0 |
| TCGA-D3-A2JN | 2022 | 1 |
| TCGA-EE-A3AG | 1265 | 1 |
| TCGA-GN-A4U3 | 3708 | 0 |
| TCGA-DA-A1I2 | 5088 | 0 |
| TCGA-EE-A3AA | 3781 | 0 |
| TCGA-EE-A3AC | 1570 | 0 |
| TCGA-D3-A1Q6 | 2184 | 1 |
| TCGA-DA-A1I1 | 5941 | 0 |
| TCGA-EE-A2MJ | 2927 | 1 |
| TCGA-EE-A2MH | 516 | 1 |
| TCGA-EE-A180 | 2889 | 1 |
| TCGA-FS-A1ZM | 3080 | 0 |
| TCGA-D3-A8GP | 4638 | 0 |
| TCGA-ER-A3ET | 2829 | 1 |
| TCGA-D9-A6E9 | 301 | 0 |
| TCGA-GN-A267 | 1960 | 1 |
| TCGA-D3-A3MU | 1209 | 0 |
| TCGA-D9-A6EG | 698 | 1 |
| TCGA-D3-A2JA | 3514 | 0 |
| TCGA-D3-A3ML | 422 | 1 |
| TCGA-DA-A1IA | 2005 | 1 |
| TCGA-BF-A3DJ | 464 | 0 |
| TCGA-D3-A3MV | 1378 | 0 |
| TCGA-EB-A4IQ | 414 | 0 |
| TCGA-WE-A8ZO | 2057 | 0 |
| TCGA-DA-A1HV | 1502 | 0 |
| TCGA-EB-A3Y7 | 326 | 1 |
| TCGA-DA-A3F8 | 492 | 0 |
| TCGA-D3-A8GL | 2711 | 1 |
| TCGA-D3-A8GB | 938 | 1 |
| TCGA-D3-A51K | 1002 | 0 |
| TCGA-DA-A3F3 | 319 | 1 |
| TCGA-ER-A2NF | 877 | 1 |
| TCGA-EE-A29E | 1940 | 0 |
| TCGA-D3-A1Q9 | 961 | 1 |
| TCGA-EE-A3JE | 1562 | 0 |
| TCGA-EE-A2GP | 423 | 1 |
| TCGA-DA-A1IC | 2071 | 1 |
| TCGA-ER-A2NG | 1490 | 1 |
| TCGA-D3-A1Q4 | 3408 | 0 |
| TCGA-EE-A185 | 151 | 1 |
| TCGA-GN-A4U7 | 317 | 1 |
| TCGA-ER-A2ND | 710 | 1 |
| TCGA-GF-A3OT | 301 | 0 |
| TCGA-D3-A51T | 818 | 0 |
| TCGA-FS-A1ZG | 295 | 1 |
| TCGA-D3-A8GD | 718 | 0 |
| TCGA-BF-A5EP | 335 | 0 |
| TCGA-XV-AAZY | 405 | 0 |
| TCGA-FS-A1Z7 | 237 | 1 |
| TCGA-WE-AA9Y | 370 | 0 |
| TCGA-D3-A5GT | 487 | 0 |
| TCGA-EB-A42Z | 441 | 0 |
| TCGA-QB-A6FS | 220 | 0 |
| TCGA-WE-A8ZR | 274 | 1 |
| TCGA-D9-A3Z4 | 519 | 1 |
| TCGA-D9-A4Z6 | 561 | 1 |
| TCGA-D3-A51H | 1714 | 0 |
| TCGA-D9-A3Z1 | 468 | 1 |
| TCGA-D3-A2J7 | 3136 | 1 |
| TCGA-GN-A4U9 | 673 | 1 |
| TCGA-D3-A2J9 | 723 | 1 |
| TCGA-GN-A26C | 821 | 1 |
| TCGA-EE-A29V | 787 | 1 |
| TCGA-WE-A8ZT | 359 | 0 |
| TCGA-DA-A1I5 | 3280 | 0 |
| TCGA-FS-A1ZH | 996 | 1 |
| TCGA-GN-A263 | 467 | 1 |
| TCGA-DA-A1I0 | 594 | 0 |

Survival status: 0 represent alive, 1represent dead.

**Table S4 Clinicopathological characteristics of melanoma patients from the training and testing group.**

| **Characteristics** |  | **Training cohort**  **(n=176)** | | **Testing cohort**  **(n=176)** | | ***P* value** |
| --- | --- | --- | --- | --- | --- | --- |
|  | **Groups** | **No.** | **%** | **No.** | **%** |  |
| age | ≤60 | 89 | 50.6 | 94 | 53.4 | 0.594 |
|  | >60 | 87 | 49.4 | 82 | 46.6 |  |
| gender | Male | 71 | 40.3 | 66 | 37.5 | 0.585 |
|  | Female | 105 | 59.7 | 110 | 62.5 |  |
| stage | I-II | 87 | 49.4 | 105 | 59.7 | 0.054 |
|  | III-IV | 89 | 50.6 | 71 | 40.3 |  |
| T | ≤T2 | 68 | 38.6 | 67 | 38.1 | 0.913 |
|  | >T2 | 108 | 61.4 | 109 | 61.9 |  |
| M | M0 | 173 | 98.3 | 170 | 96.6 | 0.502 |
|  | M1 | 3 | 1.7 | 6 | 3.4 |  |
| N | ≤N1 | 129 | 73.3 | 136 | 77.3 | 0.387 |
|  | >N1 | 47 | 26.7 | 40 | 22.7 |  |

**Table S5 Correlations between the signature and metastasis in TCGA melanoma patients****.**

| **Characteristics** | **Case** | **Metastasis** | | ***P* value** |
| --- | --- | --- | --- | --- |
|  |  | **Absent** | **Present** |  |
| All TCGA group | 352 |  |  | 0.086 |
| Low-risk group |  | 91 | 85 |  |
| High-risk group |  | 109 | 65 |  |
| Train subgroup | 176 |  |  | 0.132 |
| Low-risk group |  | 40 | 48 |  |
| High-risk group |  | 50 | 38 |  |
| Testing subgroup | 176 |  |  | 0.353 |
| Low-risk group |  | 51 | 37 |  |
| High-risk group |  | 57 | 31 |  |

**Table S6 The -2 log likelihood of our three-gene signature compared with the other three prognostic models.**

| **Prognostic model** | **-2 Log likelihood** | ***P* value** |
| --- | --- | --- |
| Risk score | 581.82 | -- |
| 7 gene signature | 593.28 | <0.001 |
| 9 gene signature | 593.98 | <0.001 |
| 10 gene signature | 596.56 | <0.001 |

**Table S7 Correlations between the expression of IGF2BP3 and clinicopathological features in melanoma patients.**

| **Characteristics** | **Case** | **IGF2BP3** | | ***P* value** |
| --- | --- | --- | --- | --- |
|  |  | **Low** | **High** |  |
| All cases | 30 | 17 | 13 |  |
| Age(years) |  |  |  | 1 |
| <60 | 10 | 6 | 4 |  |
| ≥60 | 20 | 11 | 9 |  |
| Gender |  |  |  | 0.255 |
| Male | 10 | 4 | 6 |  |
| Female | 20 | 13 | 7 |  |
| Lymph node metastasis |  |  |  | 0.017* |
| Absent | 10 | 9 | 1 |  |
| Present | 20 | 8 | 12 |  |
| Recurrence |  |  |  | 0.238 |
| Yes | 27 | 14 | 13 |  |
| No | 3 | 3 | 0 |  |
| Ulceration |  |  |  | 1 |
| Absent | 7 | 4 | 3 |  |
| Present | 23 | 13 | 10 |  |
| Tumor site |  |  |  | 1 |
| Skin | 4 | 2 | 2 |  |
| Acral | 26 | 15 | 11 |  |
| Breslow thickness (mm) |  |  |  | 0.468 |
| ≤1.0 | 3 | 1 | 2 |  |
| 1.01-2.0 | 4 | 3 | 1 |  |
| 2.01-4.0 | 12 | 6 | 6 |  |
| >4.0 | 11 | 7 | 4 |  |
| T stage |  |  |  | 0.977 |
| T1-T2 | 7 | 4 | 3 |  |
| T3-T4 | 23 | 13 | 10 |  |
| N stage |  |  |  | 0.035* |
| N0-N1 | 18 | 7 | 11 |  |
| N2-N3 | 12 | 10 | 2 |  |
| M stage |  |  |  | 0.39 |
| M0 | 27 | 16 | 11 |  |
| M1 | 3 | 1 | 2 |  |
| Grade |  |  |  | 0.068 |
| I-II | 10 | 4 | 6 |  |
| III-IV | 20 | 13 | 7 |  |

**P*<0.05
