# Supplementary figures and images for: Effects of RNA methylation N6-methyladenosine regulators on malignant progression and prognosis of melanoma
Source: Cancer Cell Int. 2021 Aug 26;21:453. doi: 10.1186/s12935-021-02163-9 (PMC8393813; doi:10.1186/s12935-021-02163-9)

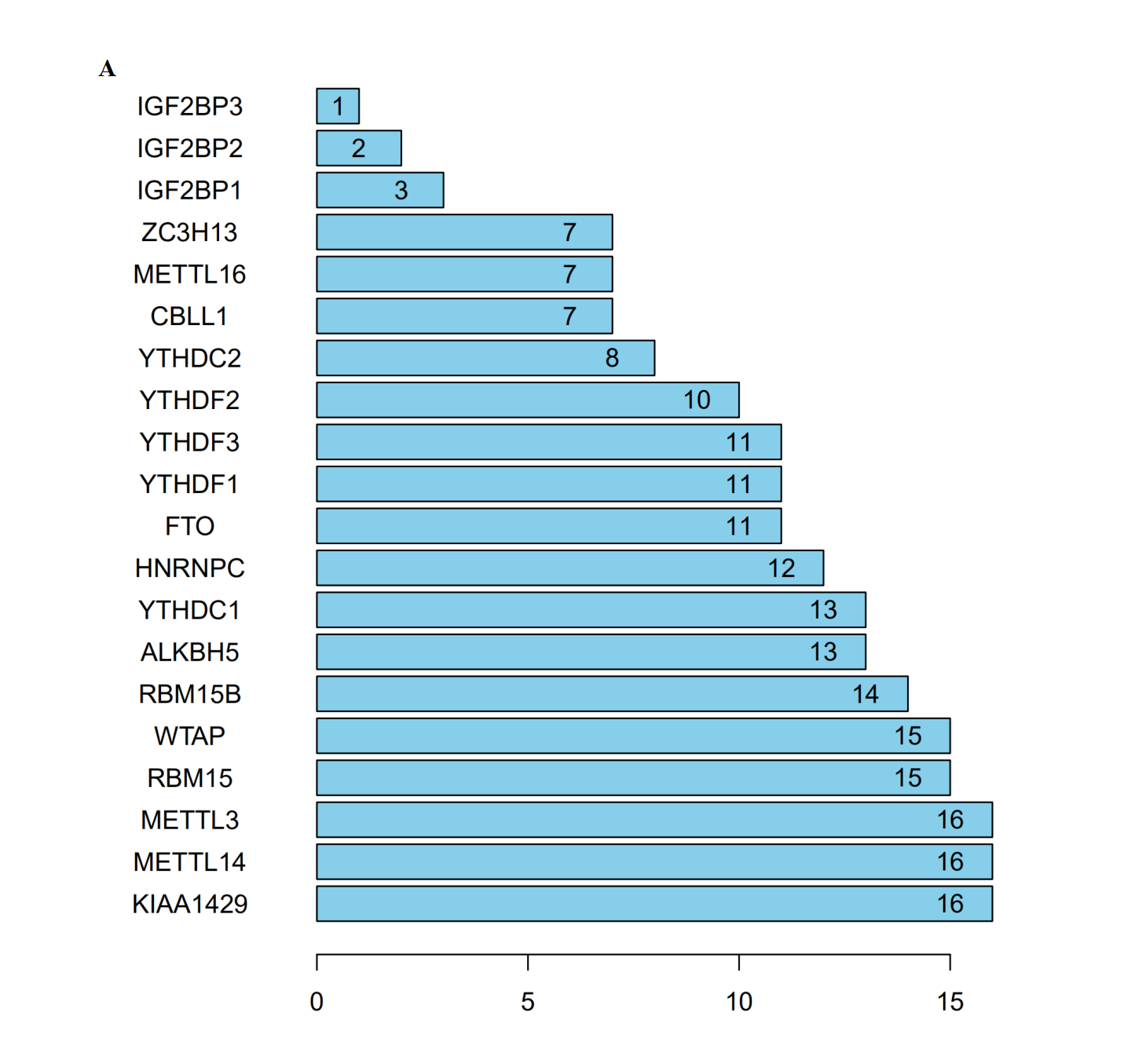

Supplement: Supplementary file 2 — Additional file 2: Figure S1. RanK of hub genes in selected twenty RNA methylation regulators. [file 12935_2021_2163_MOESM2_ESM.tif]

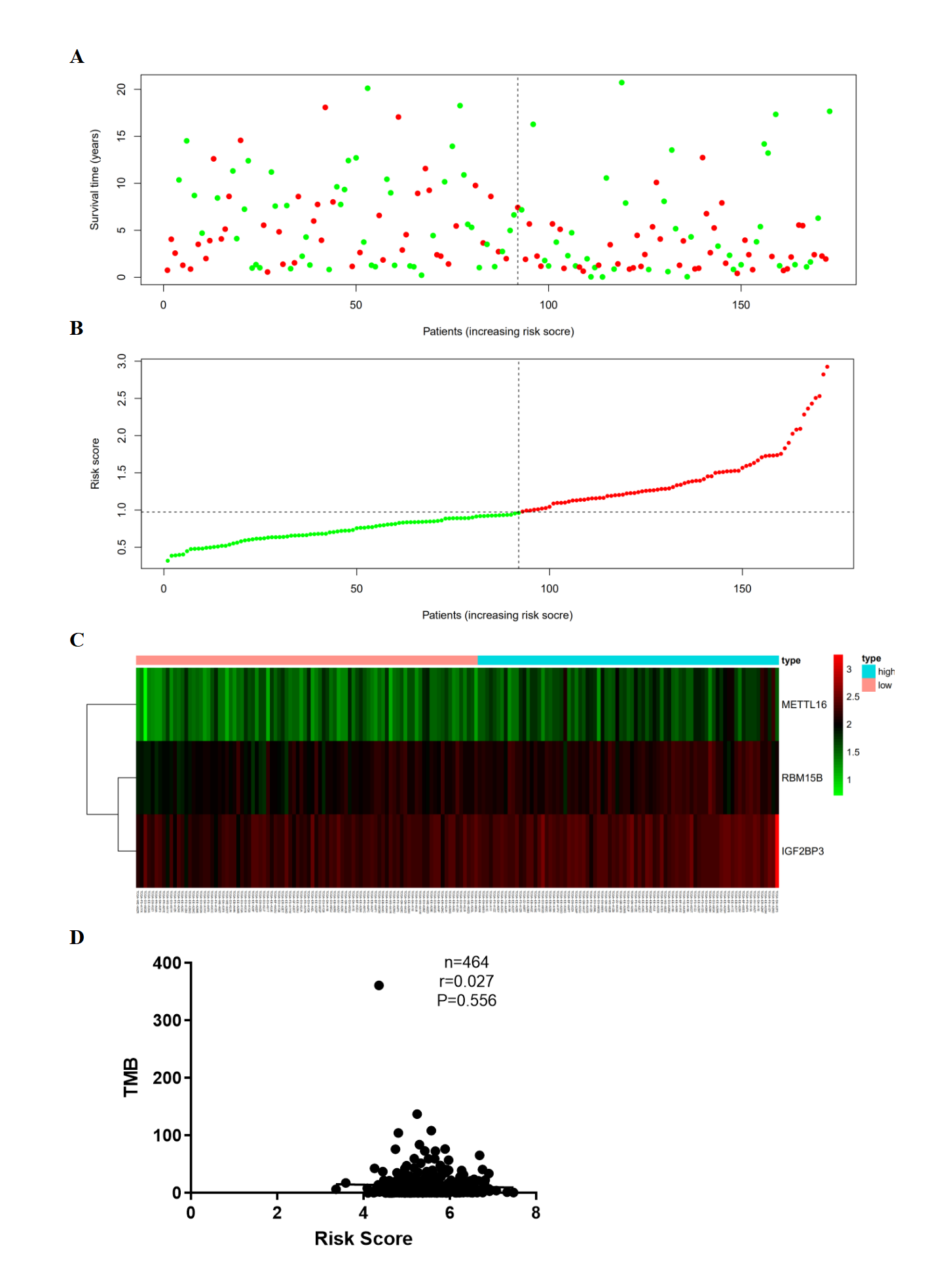

Supplement: Supplementary file 3 — Additional file 3: Figure S2. The distributions of prognostic signature-based risk scores. (A) The distributions of prognostic signature-based risk scores and their corresponding expression profiles. (B) The distributions of risk scores. (C) The distributions of risk scores and OS status. The red dots represent high-risk patients, green dots represent low-risk patients. There was no correlation between risk score and TMB in the TCGA database. [file 12935_2021_2163_MOESM3_ESM.tif]

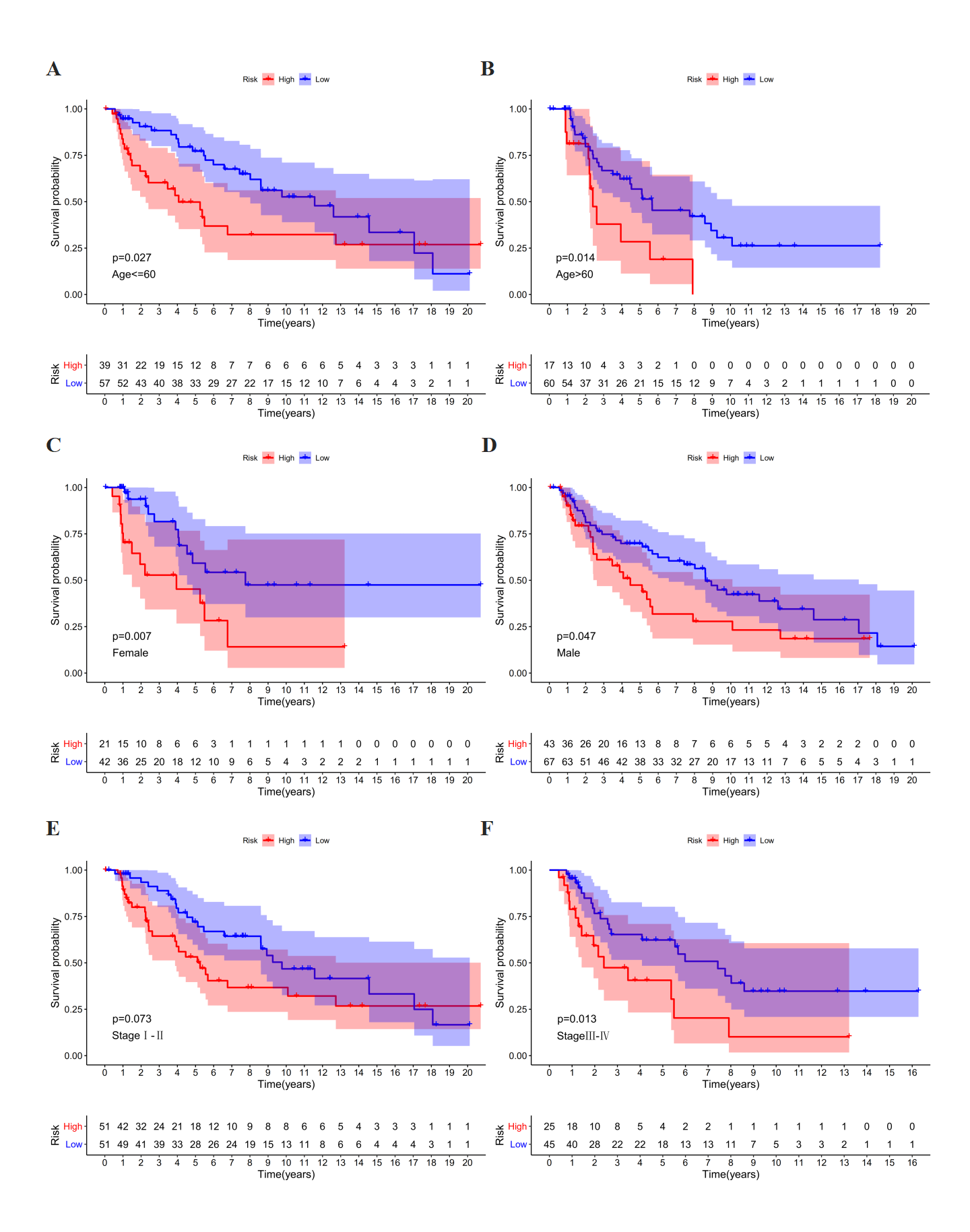

Supplement: Supplementary file 4 — Additional file 4: Figure S3. The survival analyses for the low-risk and high-risk subgroups stratified by clinicopathological parameters in the training group. (A–B) The survival analyses for the low-risk and high-risk subgroups stratified by age in the training group. (C–D) The survival analyses for the low- and high-risk subgroups stratified by gender in the training group. (E–F) The survival analyses for the low- and high-risk subgroups stratified by stage in the training group. [file 12935_2021_2163_MOESM4_ESM.tif]

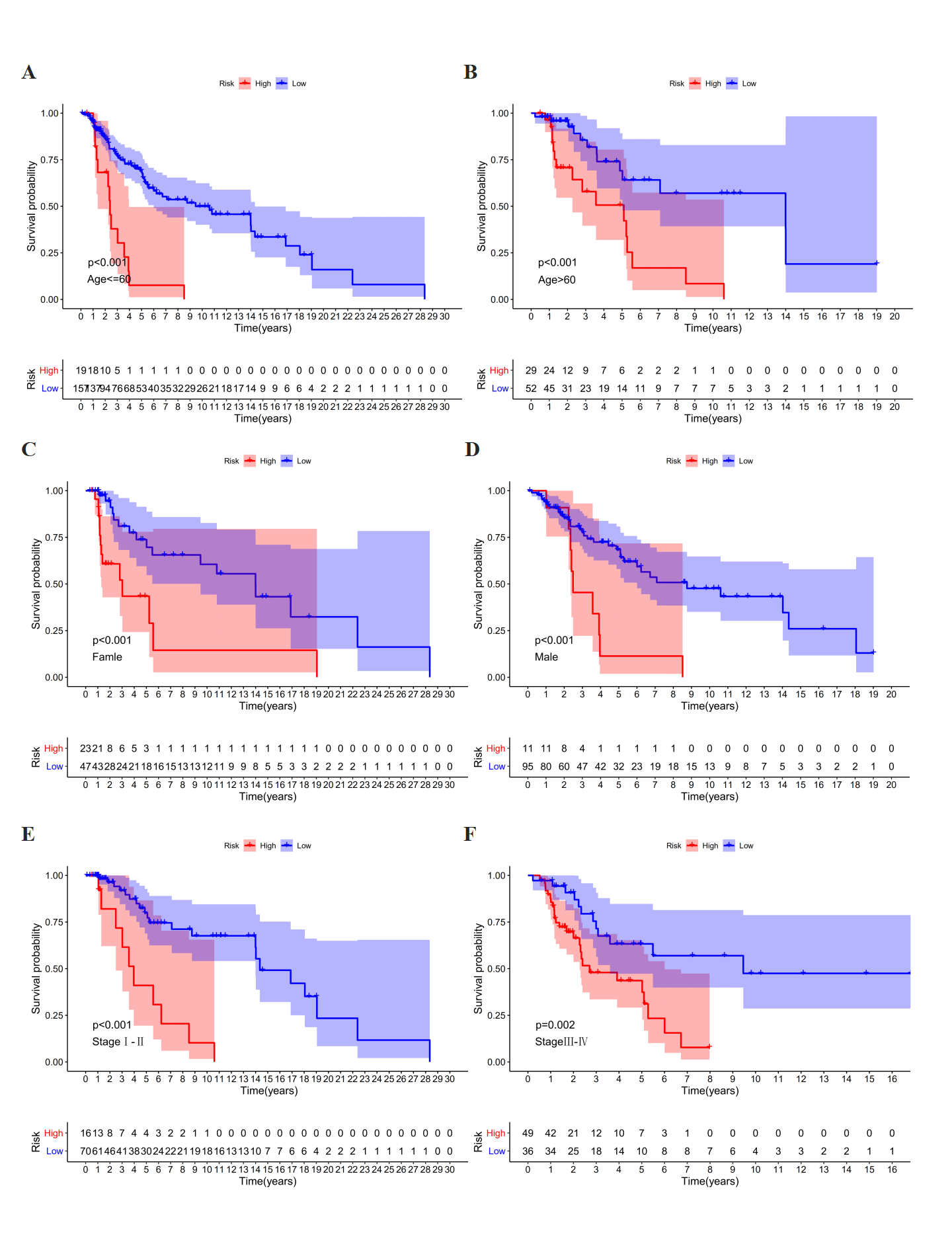

Supplement: Supplementary file 5 — Additional file 5: Figure S4. The survival analyses for the low-risk and high-risk subgroups stratified by clinicopathological parameters in the testing group. (A–B) The survival analyses for the low-risk and high-risk subgroups stratified by age in the testing group. (C–D) The survival analyses for the low- and high-risk subgroups stratified by gender in the testing group. (E–F) The survival analyses for the low- and high-risk subgroups stratified by stage in the testing group. [file 12935_2021_2163_MOESM5_ESM.tif]

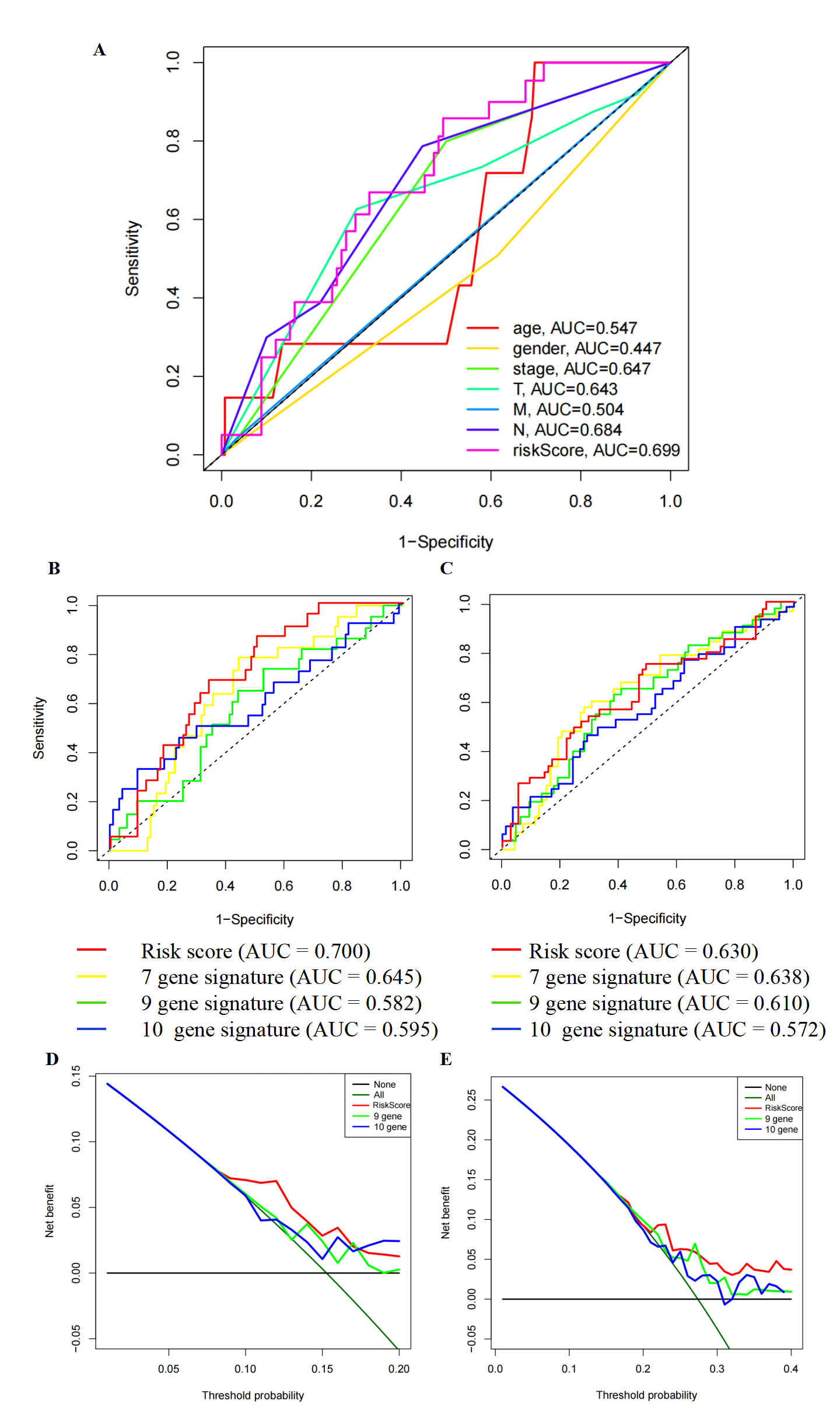

Supplement: Supplementary file 6 — Additional file 6: Figure S5. Validation of the m6A gene signature. (A) The AUC value of risk score, age, gender, clinical stage, tumor, metastasis, and node with clinical data in the training cohort. (B–C) The AUC value for 2-year and 3-year ROC of our m6A gene signature compared with other three gene-associated signatures. (D–E) The DCA curves for our m6A gene signature and other two gene-associated signatures in 2-year and 3-year. [file 12935_2021_2163_MOESM6_ESM.tif]

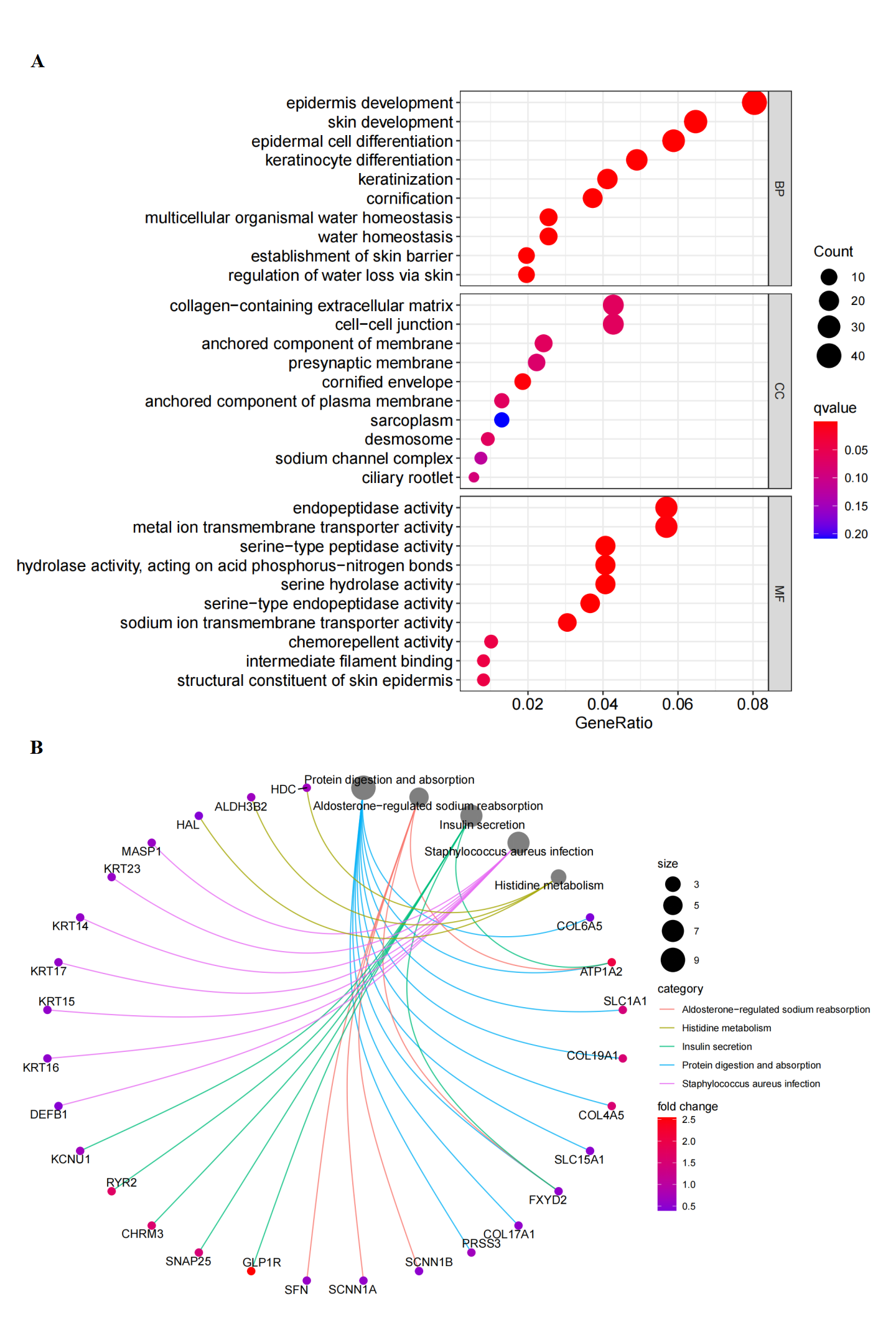

Supplement: Supplementary file 7 — Additional file 7: Figure S6. GO and KEGG pathway analysis of risk-related differentially expresses genes. (A) Bubble plots of biological process GO terms for risk-related differentially expresses genes. (B) Circle plots of biological process KEGG pathway analysis for risk-related differentially expresses genes. [file 12935_2021_2163_MOESM7_ESM.tif]

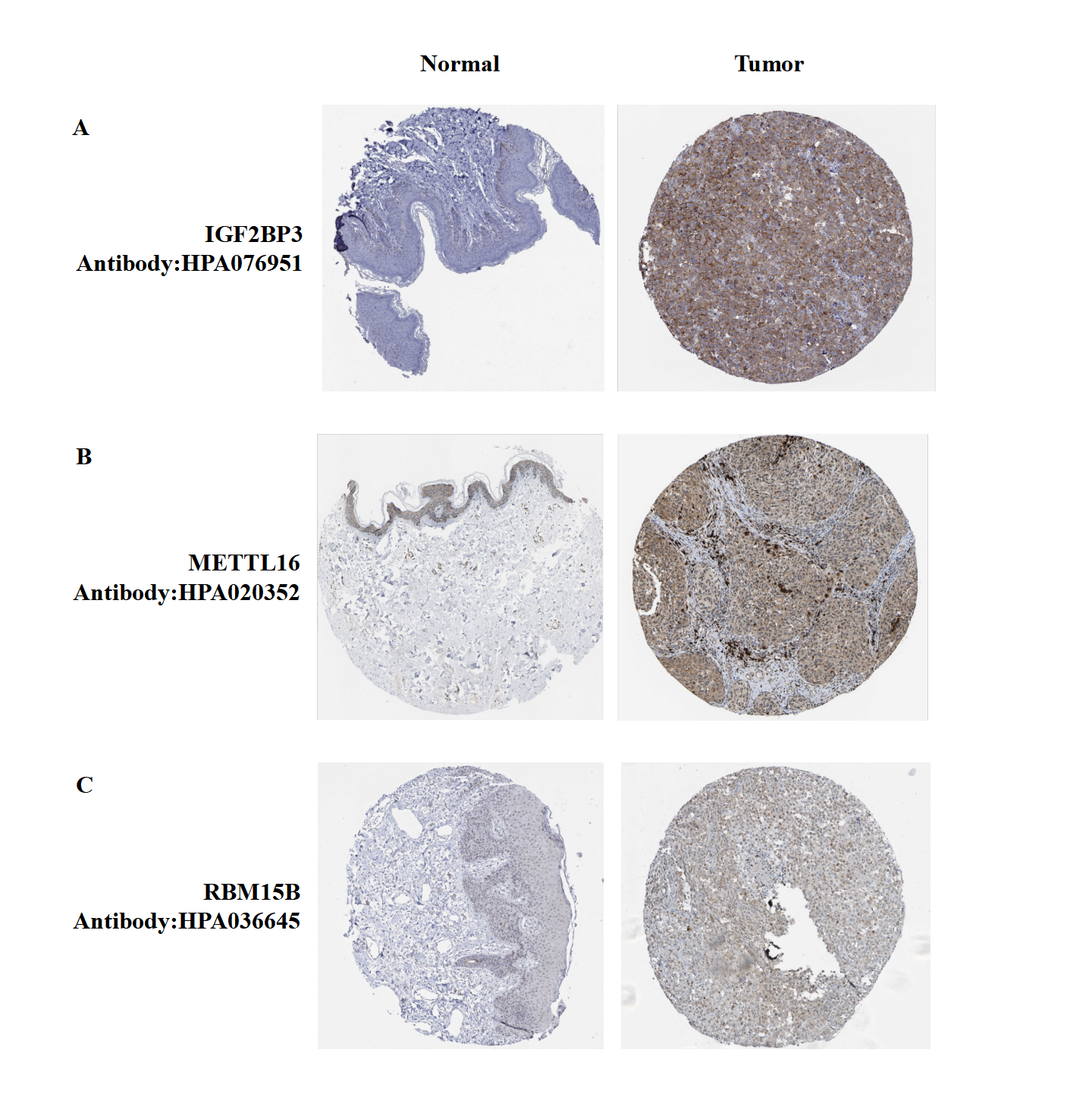

Supplement: Supplementary file 8 — Additional file 8: Figure S7. Protein expression of three hub genes detecting by an immunohistochemical assay in melanoma based on Human Protein Atlas website (www.proteinatlas.org). (A) Immunohistochemical staining showed the images of the protein expression of IGF2BP3 in melanoma. (B) Immunohistochemical staining showed the images of the protein expression of METTL16 in melanoma. (C) Immunohistochemical staining showed the images of the protein expression of RBM15B in melanoma. [file 12935_2021_2163_MOESM8_ESM.tif]

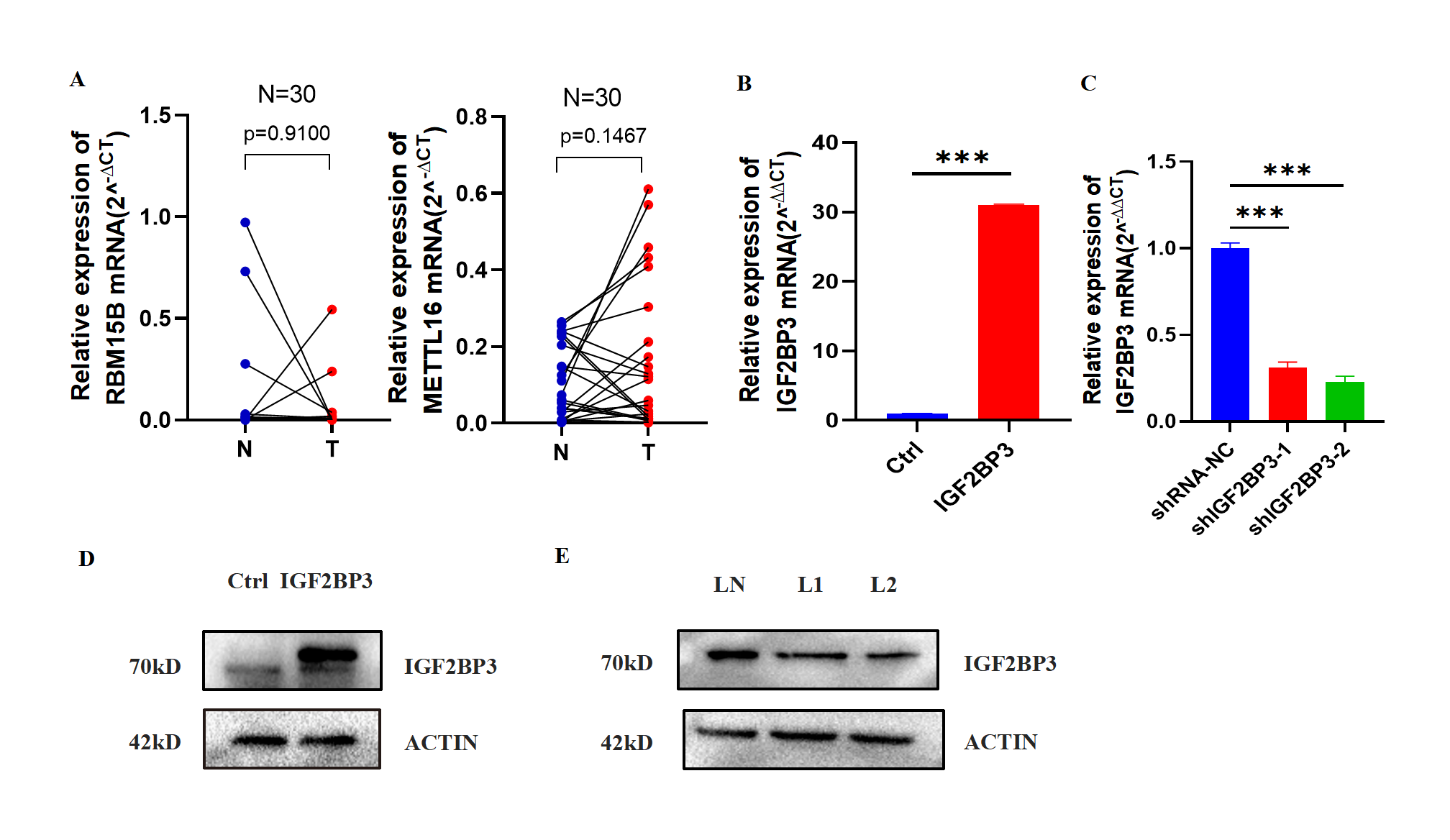

Supplement: Supplementary file 9 — Additional file 9: Figure S8. Construction of IGF2BP3 overexpressed cell lines. (A-B) RT-qPCR analysis of RBM15B and METTL16 expressions of mRNA in 30 paired fresh melanoma tissues (T) and matched adjacent normal tissues (N) quantified after transfection. (C-D) Western blot were used to confirm IGF2BP3 overexpression after transfection with lentivirus in A375 cell line. (Data are shown as the mean ± SD of three replicates. *P < 0.05, **P < 0.01, *** P < 0.001 by Student’s t-test.) [file 12935_2021_2163_MOESM9_ESM.tif]

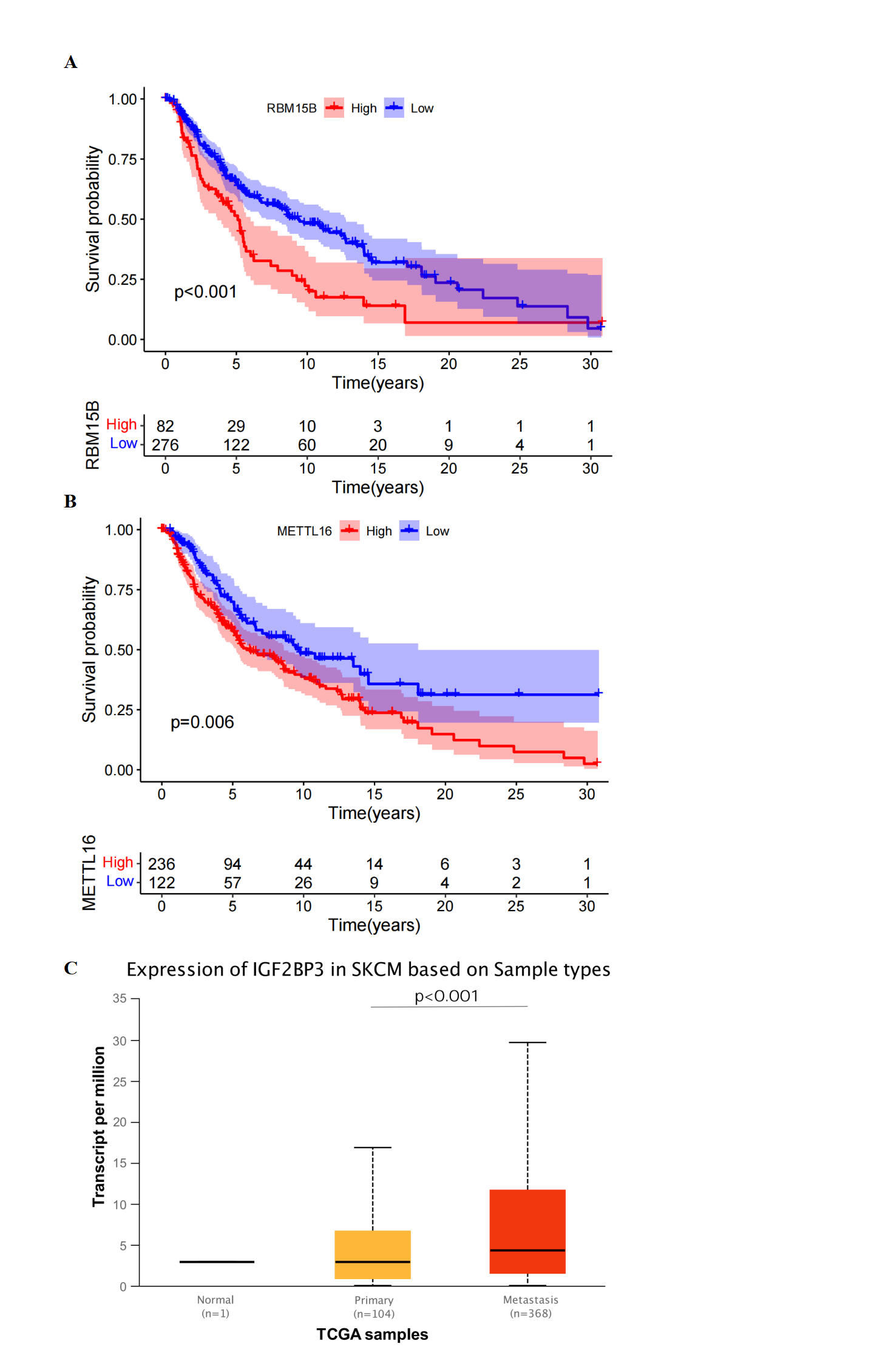

Supplement: Supplementary file 10 — Additional file 10: Figure S9. The expression levels of the risk genes in the TCGA metastasis database. (A–B) High expression of RBM15B and METTL16 were associated with poor OS in TCGA database. (C) The expression level of IGF2BP3 was verified in 1 normal tissue, 104 primary tissues, and 368 metastasis tissues from TCGA database (UALCAN). [file 12935_2021_2163_MOESM10_ESM.tif]
